# Prospective life cycle assessment of climate and biodiversity impacts of meat‐based and plant‐forward meals: A case study of Indonesian and German meal options

**DOI:** 10.1111/jiec.13549

**Published:** 2024-08-16

**Authors:** Sandra G. Marquardt, P. James Joyce, Giles Rigarlsford, Mariska Dötsch‐Klerk, Kathelijn van Elk, Jonathan Doelman, Vassilis Daioglou, Mark A. J. Huijbregts, Sarah Sim

**Affiliations:** ^1^ Department of Environmental Science, Radboud Institute for Biological and Environmental Sciences Radboud University Nijmegen Nijmegen The Netherlands; ^2^ Safety and Environmental Assurance Center, Unilever Sharnbrook UK; ^3^ Unilever Foods Innovation Centre, Unilever Wageningen The Netherlands; ^4^ PBL Netherlands Environmental Assessment Agency The Hague The Netherlands; ^5^ Copernicus Institute of Sustainable Development Utrecht University Utrecht The Netherlands

**Keywords:** biodiversity, climate change, industrial ecology, life cycle assessment, prospective life cycle assessment, scenario analysis

## Abstract

The emerging field of prospective life cycle assessment (pLCA) offers opportunities for evaluating the environmental impacts of possible future consumption shifts. One such shift involves a transition from meat‐based to plant‐forward diets, acknowledged to mitigate environmental impacts of the food system under present day conditions. Current diets are often meat intensive (“meat‐based”), whilst “plant‐forward” diets include mainly plant‐based foods, encompassing flexitarian, vegetarian, and vegan diets. Here we illustrate the application of pLCA in a case study of meal options, implementing shared socio‐economic pathway scenarios in the LCA background system to represent future production conditions. We assess the climate footprints and land‐based biodiversity footprints of a typical meat‐based meal in Germany and Indonesia compared to a plant‐forward meal in both countries (i.e., four meals), now and in 2050. Our findings show that the plant‐forward alternative maintains a lower impact per serving in all future scenarios. At the same time, the reduction in impact for the meat‐based meals is more pronounced in future scenarios due to shifts in the agricultural system. Our findings highlight the importance of supply‐side measures to produce lower‐impact ingredients, complementing demand‐side interventions to reshape food consumption. Results are further evaluated in cultural and nutritional contexts, highlighting the practical decision‐making constraints faced by consumers. We find potential “leakage” effects in calories and nutrition when choosing a lower‐impact, plant‐forward meal. These leakage effects should be considered in future studies seeking to evaluate the environmental implications of meal substitutions in the context of broader dietary requirements.

## 1 INTRODUCTION

Various population level consumption shifts have been proposed (e.g., IPCC, 2022) to address current global environmental concerns. One such shift involves a transition from meat‐based to plant‐forward diets (Willet et al., 2019). Plant‐forward diets include mainly plant‐based foods but can still include some animal products, thus encompassing flexitarian, vegetarian, and vegan diets (WBCSD, 2023). The food we eat not only influences our individual well‐being and health but, when taken in aggregate, can contribute over time to lowering environmental impacts, particularly climate, land use, and biodiversity impacts (Green et al., 2020; Kim et al., 2020; Poore & Nemecek, 2018; Sonesson et al., 2019; Springmann et al., 2018; Willet et al., 2019). Studies which investigate the future impact of the food system are valuable in guiding decision‐making, but to date have typically been carried out at a population level (e.g., Ivanovic et al., 2023; Röös et al., 2017). While population level scenarios are useful for guiding macro‐level policy, from a demand‐side perspective the decisions which matter most are individual meal selection choices.

Numerous life cycle assessment (LCA) studies have detailed the environmental impact of individual food and meal choices (e.g., Poore & Nemecek, 2018; Walker et al., 2021). However, they are anchored by the present economic background system using agricultural and energy data representing today's systems. The emerging field of prospective LCA (pLCA) offers opportunities for evaluating expected future environmental impacts of possible food and meal choices, reflecting possible changes in background economic systems. Recent developments in pLCA allow entire LCA background databases (the thousands of linked datasets which represent the background economic system) to be altered in their entirety to reflect alternative future scenarios (Beltran et al., 2020; Joyce & Björklund, 2021). To date, however, such database‐wide pLCA studies have primarily focused on transport and electricity generation (Beltran et al., 2020; Gibon et al., 2015; Hulst et al., 2020). To our knowledge, only a few pLCAs of food exist (Bohnes & Laurent, 2021; Bohnes et al., 2022). These studies are restricted to the respective foreground models (they are not “database‐wide”) and focused solely on aquaculture, coupling LCA with equilibrium modeling to identify national environmental impacts from the sector, as opposed to examining individual consumption choices.

When considering environmental impacts associated with individual food or meal choices (under current or future conditions), a vital contextual factor is the nutritional and dietary quality of the alternative choices, that is, the ability to supply adequate and balanced nutrition. Various authors observe that nutritional factors are typically not addressed in LCA studies (Drewnowski, 2005; McAuliffe et al., 2020; Sonesson et al., 2019). Where nutrition is addressed, this is typically done using mass‐based functional units (FUs) and considering individual nutrients only (e.g., protein) as opposed to being integrated in nutritional‐based FUs and/or accommodating multiple nutrients. However, Schaubroeck et al. (2018) and Sturtewagen et al. (2015) considered multiple nutrients in their assessments of meals, and there have been a few studies that have accounted for dietary quality (multiple nutrients) when comparing the environmental impacts of food systems. These have shown that the potential for impact reduction is lowered when the maintenance of dietary quality is considered a constraint (Saarinen et al., 2017; Sonesson et al., 2019). Other studies have generated theoretical diets based on food commodities optimized for both environmental impact and nutrition rather than considering the impact of actual nutritionally balanced meals (Abejón et al., 2020; Chaudhary & Krishna, 2019; Yin et al., 2020). Walker et al. (2021) found that low impact, high nutrition diets were highly monotonous, and that a trade‐off in terms of environmental impact was required to generate diets of the same nutritional quality which were palatable, met dietary requirements or were consistent with cultural norms. Indeed, when considering cultural norms, we observe that most LCA studies relating to food have focused their assessments on foods in developed countries, located primarily in Europe and North America (examples cited in McAuliffe et al., 2020; Saarinen et al., 2017; Sonesson et al., 2019). This focus neglects socio‐cultural differences in food preferences as well as differences in food and nutrition availability and security status between countries (Hallström et al., 2018; Hertwich, 2005; Kim et al., 2020). Yet, as recent studies indicate, these aspects are imperative to ensure that environmentally preferable ingredient and/or meal suggestions align with peoples’ needs and preferences (Arrieta et al., 2021, 2022).

In this paper, we assess the current and future change in environmental impact of moving from an actual meat‐based to a plant‐forward meal in two distinct cultural settings. Specifically, we assess the climate and land‐based biodiversity footprints of a typical meat‐based meal in Germany and Indonesia compared to a plant‐forward meal in both countries (i.e., four meals), now and in 2050. Future environmental impacts are assessed by utilizing the Futura framework, a database‐wide pLCA framework developed by Joyce and Björklund (2021), to implement shared socio‐economic pathway (SSP) scenarios (Bauer et al., 2017; O'Neill et al., 2017; Riahi et al., 2017) in the LCA background system (see Section 2.1.1). In addition, to evaluate the dietary quality of the meal options, we benchmarked the environmental footprints against the meals’ respective Nutrient Rich Food (NRF) 15.3 index scores and indispensable amino acids (IAA) score (a measure of protein completeness).

By looking at real‐world meal substitutions in two very different food cultures within a pLCA framework, we investigate the following questions:

- How does the expected future environmental impact of real‐world meal substitutions change under different SSP scenarios and in distinct food cultures?
- What are the potential nutritional constraints associated with these substitutions and how might this affect our interpretation of the environmental impact?

## 2 METHODS

### 2.1 Prospective life cycle assessment

**FIGURE 1:**
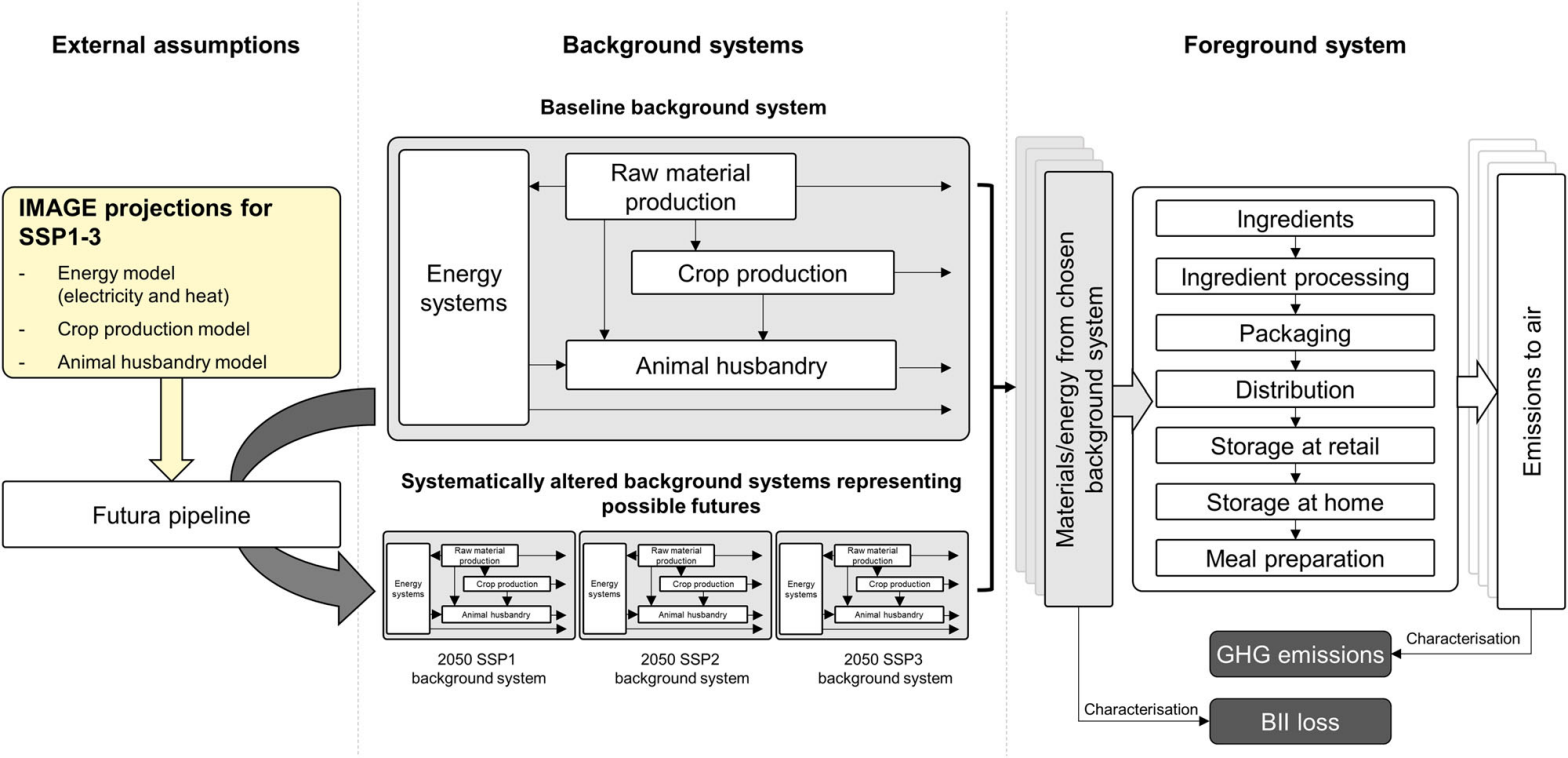
Diagram outlining the conceptual framework for the environmental assessment of meal options. IMAGE projections for shared socio‐economic pathway (SSP) 1−3 were applied to the baseline background system via Futura (Joyce & Björklund, 2021) to create alternative SSP1−3 background systems. Each of these background systems was then used in conjunction with the foreground system defined for one serving of each meal to generate estimates of biodiversity loss and greenhouse gas (GHG) emissions per serving, for each meal‐scenario combination.

A pLCA approach was used in this study. We applied the Futura pLCA framework (Joyce & Björklund, 2021) to develop comprehensively updated background databases reflecting changes to energy and agricultural systems in 2050 against which foreground models of specific meal choices could be assessed. We generated background systems representative of three possible scenarios for 2050 (Figure 1), based on projected scenario results for three of the five SSP scenarios (Bauer et al., 2017; O'Neill et al., 2017; Riahi et al., 2017), chosen to represent best (SSP1), reasonable (SSP2), and worst‐case (SSP3) assumptions. Futura was chosen as this open‐source software allows a simple and consistent approach to applying the SSP scenarios to background LCA databases (Ecoinvent and World Food Life Cycle Database [WFLDB]). Futura consistently generates local versions of altered background databases according to a set of predetermined steps. This means that given the same original databases and transformation steps, any practitioner can generate an identical copy of the future databases used in this study. The foreground models for our case study meals are modeled separately and linked to the altered background databases (Figure 1). The background databases could, in theory, be used to model any product produced in that future scenario. This study represents the first major application of the Futura framework, and its first implementation in the agri‐food sector.

The SSP scenarios were originally developed to complement climate change scenario modeling by providing a range of different socio‐economic development options and offer distinct outlooks on future global development trajectories. SSP1, known as “Taking the Green Road,” prioritizes sustainability. SSP2 represents a “Middle of the Road” approach (based on a continuation of “business as usual”). SSP3, dubbed “A Rocky Road,” is characterized by regional rivalry. Key overall drivers of the SSPs include human population size, shifts in gross domestic product, and levels of urbanization, which align with each SSP's narrative. These drivers are further influenced by factors which are particularly relevant in this context, such as food consumption patterns (e.g., low animal‐based consumption in SSP1, high in SSP3), agricultural productivity gains (high in SSP1, low in SSP3), transition to renewable energy (high in SSP1, low in SSP3), and international trade orientation (global integration in SSP1, protectionism in SSP3). As a result, the three scenarios diverge in projected agricultural supply and demand, production intensities, energy carrier dynamics, and land use patterns.

Data to implement the SSP scenarios above was taken from the integrated assessment model IMAGE (Stehfest et al., 2014). The IMAGE modeling framework integrates various sub‐models representing socio‐economic processes such as the food and energy systems, as well as biophysical systems such as land use and land use change, the carbon cycle, and climate dynamics. The scenario results from two IMAGE model components were particularly relevant for our implementation of the pLCA: the TIMER energy system recursive dynamic simulation model (van Vuuren, 2007) and the coupled agro‐economic model MAGNET and land use IMAGE‐Land Management for land system dynamics (Doelman et al., 2018; Woltjer et al., 2014). Results were available for 26 regions, 30 electricity technologies, 16 crops, and 5 livestock systems.

In order to implement the SSP scenarios in the LCA background system we focused on three components: electricity technology and grid mix, crop production, and animal husbandry. As such, we were able to build on the work for electricity by Beltran et al. (2020) and extended the approach to include agricultural systems, that is, those which are most likely to affect the results of LCAs of food. We identified agricultural background LCI datasets in the ecoinvent and WFLDB databases and mapped them to the appropriate scenario data from the IMAGE results (see Supporting Information S1 Section 1 for details). Second, we implemented changes to the background system as follows: changing input–output structure of the production system indicating a change in technical efficiency and changes in market mix of products for a region.

For electricity, the LCA background system data originated from the ecoinvent 3.5 cut‐off unit process model. We altered the technical efficiency of electricity production systems by scaling energy carrier inputs (e.g., coal, gas) using the scenario‐ and region‐specific efficiency changes projected by the IMAGE model. Furthermore, using the production volumes for different technologies projected by IMAGE, we derived region‐specific electricity grid mixes to update ecoinvent's electricity mixes (see Supporting Information S1 Section 2 for mapping).

For food and feed crop production activities, we implemented efficiency changes at the output level. Scaling at the output level assumes that the efficiency of all inputs to crop production alters using the same factor. While changes in yield could be triggered by changed efficiency of only selected inputs (e.g., driven by changed fertilizer efficiency while irrigation efficiency remains unaltered), IMAGE does not supply sufficiently granular information to differentiate efficiencies at this level. We did not alter the market mix of crops (e.g., relative share of production systems such as irrigated vs. rain fed) in the background system as the information required to do this was not available for the regional inventory datasets.

For animal husbandry, we accounted for changes in feed conversion efficiency and changes in feed basket composition. Feed efficiency changes from IMAGE were used to scale feed intake, manure management, and enteric emissions (all implemented as “inputs” in the relevant datasets). We included manure management and enteric emissions in this adaptation since both are directly related to feed intake (Nemecek et al., 2019). Feed basket composition indicates the relative share of different feed (hay, fodder, etc.) used in the livestock systems and was also projected by IMAGE. Data from IMAGE had insufficient granularity of livestock systems to also implement changes in the market mix (i.e., the relative share of different livestock systems for each region).

### 2.2 Case study

#### 2.2.1 Product system and function

The product systems that we consider in the case study consist of “meat‐based” and “plant‐forward” variations of main meals that are culturally relevant in one developed nation (Germany) and one developing nation (Indonesia). The four meals selected for this study are entire, balanced meals, containing ingredients from all the major food groups. “Meat‐based” and “plant‐forward” are defined with respect to the main element of the meal. As such, the plant‐forward variations consist of a plant‐based main element, but are not necessarily entirely plant‐based, primarily due to cultural and nutritional constraints. The meals are already popular and widely consumed within the chosen countries, promoted through the Knorr and Royco brands. We use the NRF15.3 index and IAA to assess the nutritive value of the meals (see Section 2.2.5).

For Germany, the meal assessed is spaghetti bolognese, with a beef version for the meat‐based variation and a lentil version for the plant‐forward variation. This meal aligns with the needs of younger, primarily urban people living in small households in Germany, who have been identified as willing to experiment with non‐meat alternatives (Koch et al., 2019). The recipe consists of an identical tomato‐based sauce, containing either lentils or ground beef, and served with the same amount of pasta. The meal recipes were taken from on‐pack recipes given on Knorr Fix seasoning spaghetti bolognese “mealmaker” products sold in Germany.

For Indonesia, the meals considered are composites, based around a soup. They consist of a soup, rice, side dish, and fruit. The meal suggestions have been formulated according to the Indonesian Balanced Nutrition Guidelines, using the “my plate guide” (Kodyat, 2014). Malnutrition, particularly in children, is an ongoing concern in Indonesia (WHO, 2017). Thus, recipes have been primarily designed to provide affordable and nutritionally balanced meals using easily available ingredients with high consumer acceptance. For the meat‐based variant, a chicken soup provides the main protein source, accompanied by rice, fried beans, and banana taken from the Royco Nutrimenu website (Royco, 2020). For the plant‐forward variant, the main protein source is a tofu soup, accompanied by rice, an omelette with chicken and beansprouts, and watermelon based on meals given on the Royco Nutrimenu website. Inclusion of animal products (chicken and egg) in the plant‐forward variant is intended to ensure the nutritional quality of this meal by using affordable, widely available, and culturally acceptable ingredients.

#### 2.2.2 Functional unit

The function of a meal is multifaceted and context dependent, consisting of social, energetic, and nutritional elements. This presents an interesting challenge in setting a suitable functional unit within LCA. Here, two separate functional units are defined, broadly representing the social and energetic axes, while an adjacent analysis is performed to consider the nutritional aspect. The function of a meal can be broadly described as “being the food that you eat at a given mealtime.” This is likely to be the most relevant generic function from a consumer perspective, and as such, the unit that will be most relevant for individual consumer decision‐making. Provided the collection of food items within a recipe can be objectively considered to be a meal, the first functional unit is therefore described as “the provision of one prepared serving of a meal.” In order to account for the energetic aspect of the function of a meal, a second functional unit is defined as “the provision of 100 kcal of energy by a prepared meal.” Here the reference flow is the amount of each of the elements of the meal (taken in aggregate) which would be required to deliver 100 kcal. The more energy dense the meal, the lower the amount of food required to meet this functional unit.

Several authors have investigated the use of “nutritional functional units” based on composite nutritional indicators for LCA studies of food (reviewed in McAuliffe et al., 2020). However, we do not adopt this kind of nutritional functional unit in our study, choosing instead to consider nutritional quality in a separate analysis. We do this partly because the meals in scope have been specifically designed as balanced meals and, in the Indonesian case, align with national nutritional guidelines. In addition, we note that the usefulness of composite nutritional indicators, such as NRF scores, within functional units is compromised by the difficulties associated with translating such an indicator specified in a functional unit to a suitable reference flow. For example, in this study, as the composition of the meals is determined by the recipe, the reference flow of each of the meals cannot be adjusted to deliver a given NRF score. In cases where these indicators include negative scores for disqualifying nutrients (e.g., NRF9.3, NRF15.3, Nutrient Quality Index), this can also lead to perverse results, especially for individual foods obligately high in such nutrients, for example, fats and oils (see Sonneson et al., 2019).

#### 2.2.3 Impact assessment methods

Two impact categories are considered in this study, climate change and biodiversity loss due to land use. These have been defined within the planetary boundaries (PB) concept as “core” PB categories—“each with the potential on its own to drive the Earth system into a new state should they be substantially and persistently transgressed” (Steffen et al., 2015). They are also particularly relevant impact categories for the food sector as “global food production threatens climate stability and ecosystem resilience [and] constitutes the single largest driver of environmental degradation and transgression of planetary boundaries” (Willet et al., 2019). Climate change was quantified using IPCC AR5 Global Warming Potentials with a time horizon of 100 years (IPCC, 2013). For biodiversity integrity, we used land‐based Biodiversity Intactness Index (BII) loss factors (Supporting Information S1, Table S1) based on Newbold et al. (2016). This indicator reflects the loss in biodiversity intactness (i.e., the loss in local abundance corrected for differences in species’ occurrence) between different land use types and natural vegetation (Newbold et al., 2016).

#### 2.2.4 System boundaries and life cycle inventory

A cradle‐to‐grave approach was taken for the LCA, encompassing the agricultural production of ingredients and their processing to the point of purchase, the raw materials and processing required for the packaging of these ingredients, distribution and storage (both at retail and in home), and the energy used in the preparation of the meal (Figure 1). Disposal of packaging was excluded from the assessment (Supporting Information S1, Table S2).

Recipes and key assumptions for the modeling of the individual life cycle phases of the meals, prepared and consumed in home, are outlined in Tables S3 to S6 (Supporting Information S1). Background LCI data were primarily sourced from the ecoinvent 3.5 database using the cut‐off system model (Wernet et al., 2016). This was complemented by data from the WFLDB 3.5 for agriculture and food products (Nemecek et al., 2019). Data gaps and additional datasets were filled using information from Poore and Nemecek (2018), Agribalyse v3.0 (Asselin‐Balençon et al., 2020), and information from the literature (see Supporting Information S1, Table S4 and S6).

#### 2.2.5 Nutritional assessment

We calculated the NRF 15.3 index scores for the meal options (Fulgoni et al., 2009) using nutritional information obtained from the German Nutrient Database (Bundeslebensmittelschlüssel or BLS) (Hartmann et al., 2014) for Germany and, in the absence of an appropriate national database, from the United States Department of Agriculture (USDA) nutrient database (USDA, 2019) for Indonesia. Details of the nutritional assessment, including the nutritional data for each meal can be found in Section 7 of the Supporting Information S1. The NRF15.3 reflects nutrient density of food and is frequently used in nutritional assessments and combined with environmental assessments (Hallström et al., 2018; Mertens et al., 2017; Sonesson et al., 2019; Van Kernebeek et al., 2014). It accounts for 15 qualifying nutrients (dietary fiber, protein, potassium, calcium, iron, zinc, folate, Vitamin A, Vitamin D, Vitamin E, Vitamin B1, Vitamin B2, Vitamin B12, Vitamin C, and monounsaturated fat) and three disqualifying nutrients (saturated fatty acids, sugar, and sodium) (Fulgoni et al., 2009). While studies have shown that including up to 10 qualifying nutrients provides sufficient results in terms of the nutritional quality of diets (Fulgoni et al., 2009), additional nutrients might be at risk in the context of moving to more plant‐based diets (Willet et al., 2019). We therefore chose to use the NRF15.3 over other scores as it includes these specific nutrients (iron, calcium, zinc, Vitamin B12, Vitamin B2, and Vitamin D) (Fulgoni et al., 2009; Willet et al., 2019).

Further, as an additional nutritional assessment, we looked at protein completeness using the IAA score, defined as the amount of the most limiting IAA in a food's protein relative to the recommended amount of this IAA in a reference protein. IAA scores of ≥100 indicate that a protein has a complete IAA pattern, while lower score indicates that at least one IAA falls short (WHO, 2007).

## 3 RESULTS

### 3.1 Baseline results

**FIGURE 2:**
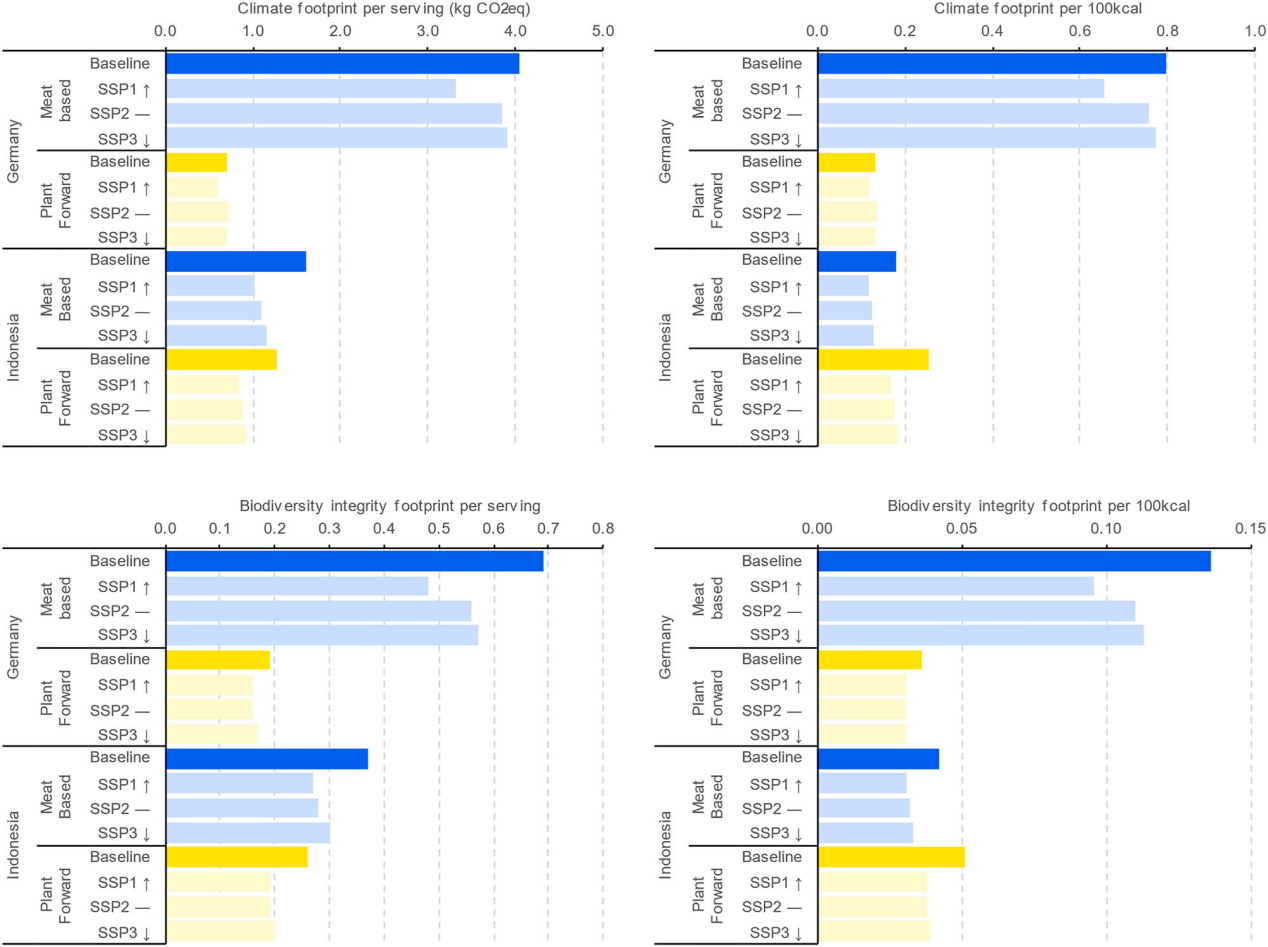
Climate (top) and biodiversity (bottom) impacts of each of the four meals assessed in this study across the baseline and three future scenarios modeled in the prospective life cycle assessment (shared socio‐economic pathway 1 to 3). Results per serving are shown in the left two panels and results per 100 kcal are shown in the right two panels. Underlying data for this figure are available in the corresponding tab in Supporting Information S2 Excel file.

The climate and biodiversity impacts per serving and per 100 kcal for the base year are shown in Figure 2. Across the four meals and for both metrics, the German meat‐based meal (beef bolognese) consistently has the highest impact, while the German plant‐forward meal (lentil bolognese) consistently has the lowest. As a result, we see a substantial reduction in impact associated with a potential switch from a meat‐based to plant‐forward version of this meal in Germany. In Indonesia, however, the pattern is not so straightforward. First, the absolute difference in impact between the baseline plant‐forward and meat‐based meals is smaller than that seen in Germany. In addition, for both metrics, when the impacts are considered per serving the meat‐based variant has the higher impact, however when considered per 100 kcal the plant‐forward variant has the higher impact. This is primarily because the meat‐based Indonesian meal is a higher calorie meal compared to the other three meals considered, providing 44% of the recommended daily intake of calories, while the other meals each provide 25%–26%.

### 3.2 Future scenarios

#### 3.2.1 Germany

In all future scenarios, both the climate and biodiversity footprints of the German meat‐based meal were lower than respective baseline footprints. The largest reduction in impact is seen in the most ambitious (SSP1) scenario (an 18% reduction in climate impact and 29% reduction in biodiversity impact) (Figure 1). The reduction in both impacts is primarily driven by changes in animal husbandry practices affecting the efficiency, and therefore the impact, of beef production. Increases in efficiency of crop production, both as feed for cattle and in the production of the pasta, also contribute to lowering the footprint of the future scenarios. Both the climate and land‐based biodiversity impacts of the meat‐based meal in Germany remain substantially higher than the plant‐forward variant across all three future scenarios.

#### 3.2.2 Indonesia

The climate and biodiversity footprints for both Indonesian composite meals are lower than the baseline across all three future scenarios and are consistently lowest in SSP1, followed by SSP2 and then SSP3. The magnitude of the reduction is similar for both meal types for both impacts across all scenarios (Figure 1). For climate, the reduction is slightly lower for the plant‐forward meal than the meat‐based meal (e.g., 34% reduction vs. 36% reduction, respectively, for SSP1), whereas for biodiversity the reduction seen in the plant‐forward meal is slightly higher (27% reduction vs. 26% reduction, respectively, for SSP1). For climate, the primary driver of the reduction in impact is the lower GHG intensity of energy that is seen across all three scenarios for Southeast Asia. For biodiversity, the differences seen are solely driven by the increased land efficiency in plant production, primarily poultry feed in the value chain of the eggs and chicken meat and the soybeans in the value chain of the tofu.

### 3.3 Drivers of impact

#### 3.3.1 Germany

**FIGURE 3:**
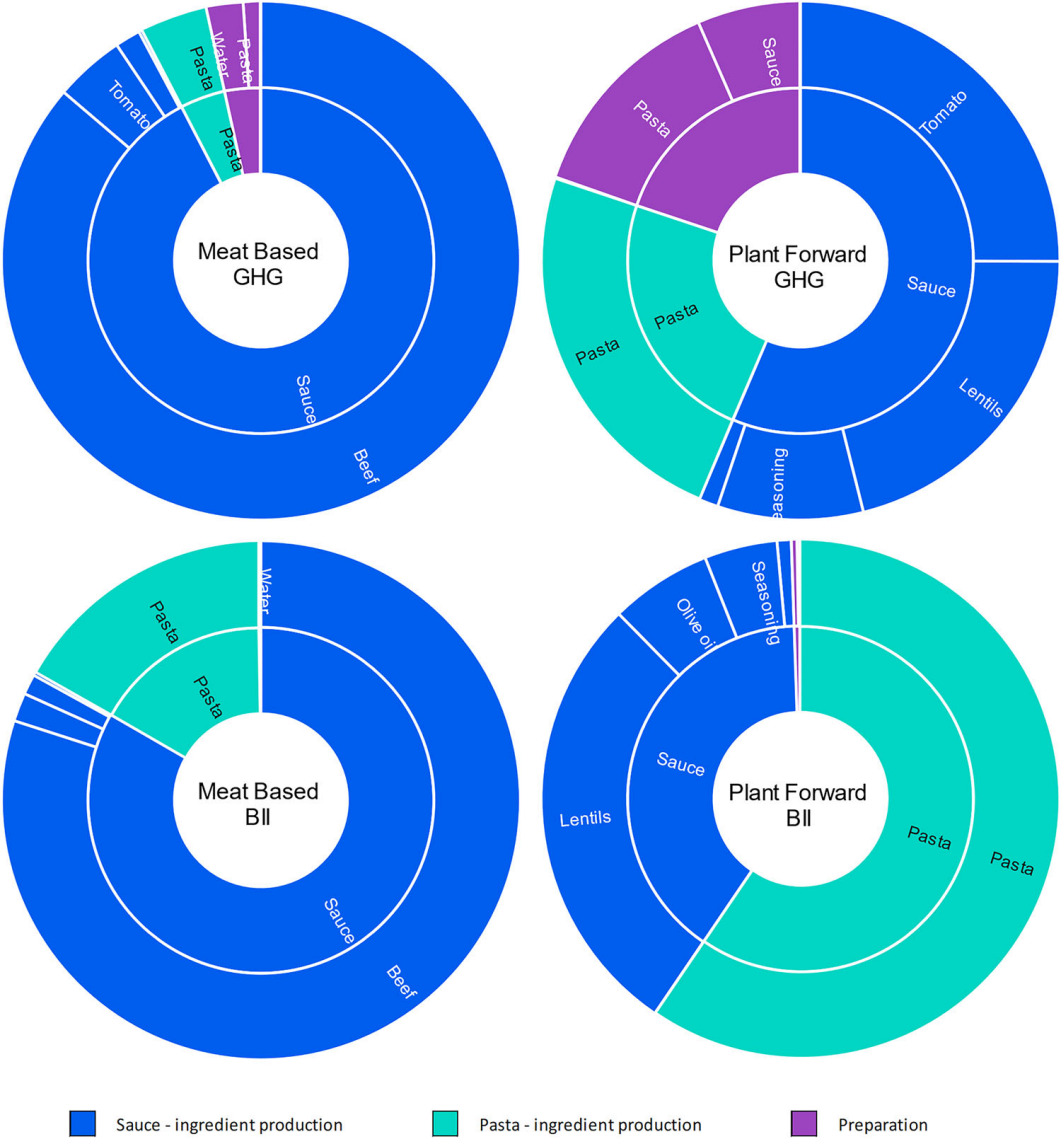
Relative climate and biodiversity impact contribution of meal components and preparation stage (inner ring) and each of the constituent parts of those meal components/preparation (outer ring, segments of the same color) for the German meals in the baseline scenario. Note: while the total impact when considered per serving and per 100 kcal differs (Figure 2), the relative proportions of impact of each of the components are identical for both functional units. Underlying data for this are available in the corresponding tab in Supporting Information S2 Excel file.

The climate and biodiversity impact of the German meat‐based meal is overwhelmingly driven by the beef in the baseline year (Figure 3 ) and in all future scenarios. The absolute impact of the non‐beef components remains broadly constant across the future scenarios, thus as the impact of beef decreases in the future (most markedly in SSP1), the relative contribution of the remaining components increases. In all scenarios, however, the beef remains the primary driver of the impact of the meal. When lentils are used as the main source of protein in the plant‐forward variant, the impact of that part of the meal is far lower (21% climate, 28% biodiversity), so the relative contribution of the other ingredients and of the preparation of the meal is greater.

#### 3.3.2 Indonesia

**FIGURE 4:**
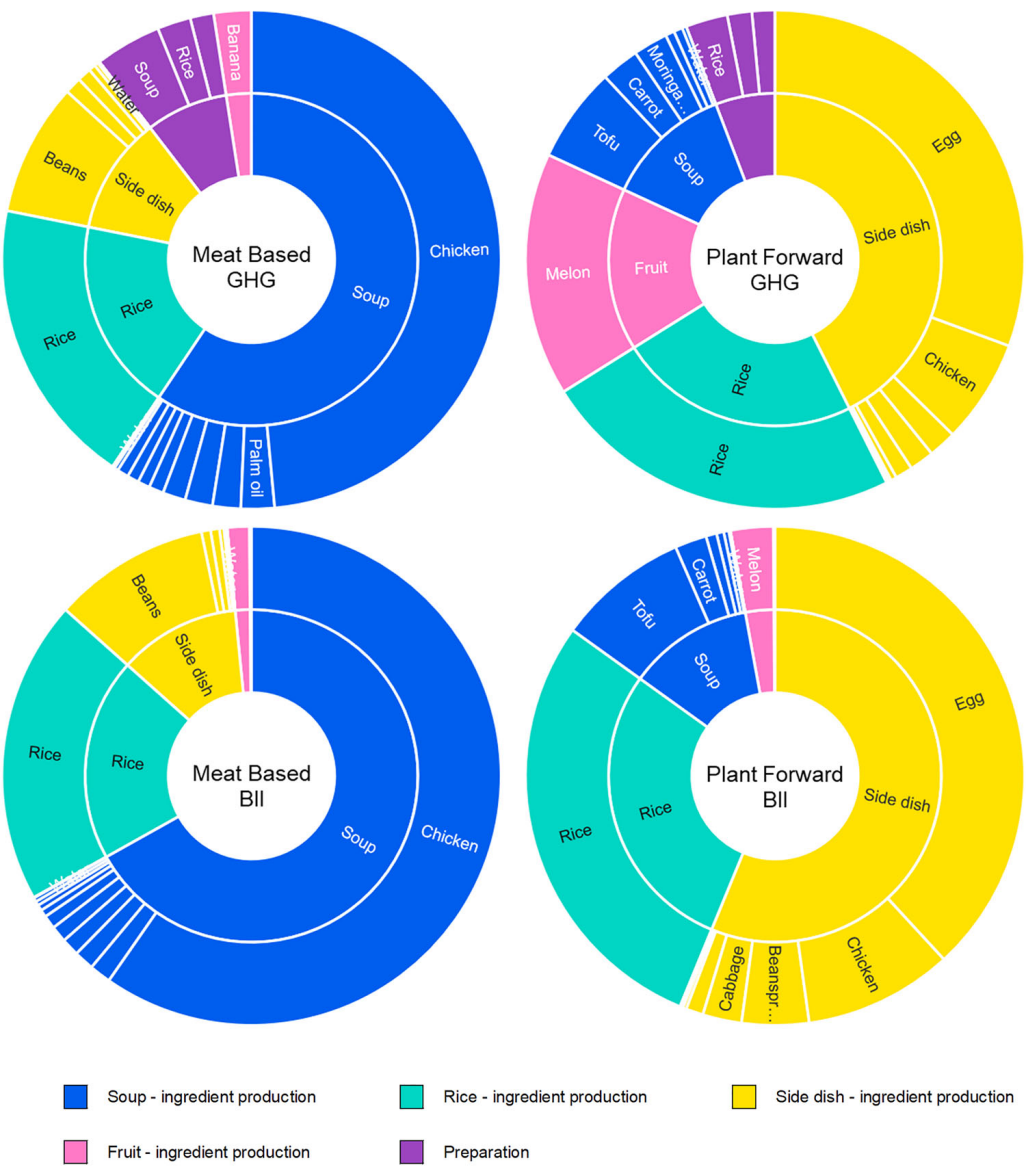
Relative climate and biodiversity impact contribution of meal components and preparation stage (inner ring) and each of the constituent parts of those meal components/preparation (outer ring, segments of the same color) for the Indonesian meals in the baseline scenario. Note: while the total impact when considered per serving and per 100 kcal differs (Figure 2), the relative proportions of impact of each of the components are identical for both functional units. Underlying data for this figure are available in the corresponding tab in Supporting Information S2 Excel file.

For the Indonesian meals, the protein components represent the largest contribution to climate and biodiversity impact (Figure 4). For the meat‐based meal this is the chicken in the soup, while for the plant‐forward meal it is the egg in the omelette which is the primary driver of climate and biodiversity impact, with the chicken (in the omelette) and the tofu (in the soup) also noticeable contributors to impact. In addition, the choice of watermelon in the plant‐forward meal has a substantially higher climate impact than the banana in the meat‐based variant.

### 3.4 Nutritional assessment

The nutritional parameters calculated for each of the meals considered are shown in Table 1.

**TABLE 1:** Nutritional quality of meals.

			NRF15.3		
		kcal per serving	Per serving	Per 100 kcal	IAA
Germany	Meat‐based	508	319	63	108
	Plant‐forward	529	208	39	86
Indonesia	Meat‐based	884	593	67	107
	Plant‐forward	505	349	69	159

In both Germany and Indonesia, the plant‐forward meals have a lower NRF15.3 index score than the meat‐based meals. For the German meals the difference in per serving NRF15.3 score is driven by the difference in Vitamin B12 (accounting for 100 out of the 111 point difference). In terms of protein completeness for the German meals, the IAA score of the meat‐based meal was above 100. The plant‐forward meal had an IAA score of 86, with lysine the limiting amino acid. Both German meals provide a similar number of calories.

**FIGURE 5:**
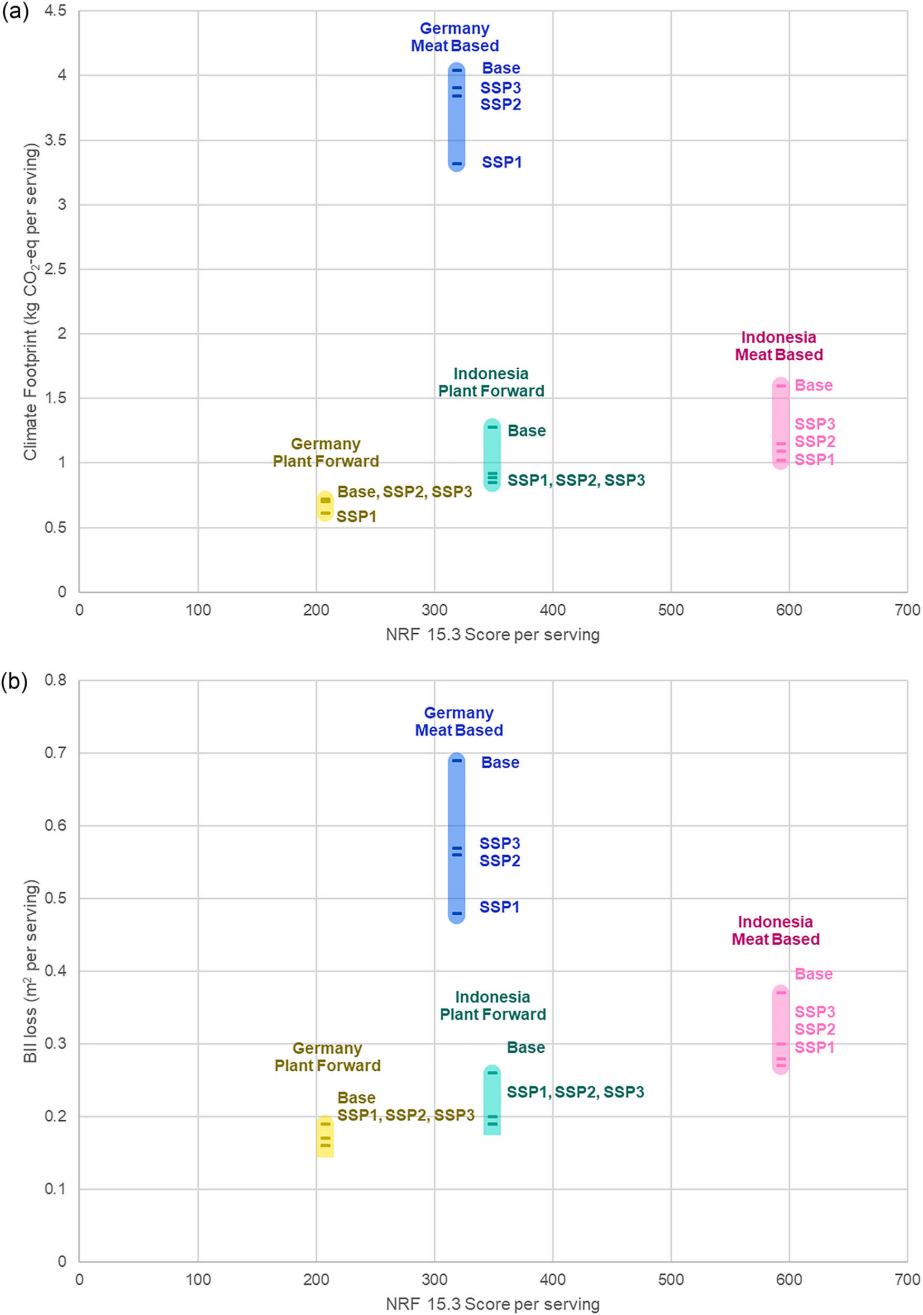
Climate impact (a) and biodiversity impact (b) per serving plotted against NRF15.3 index per serving for each of the four meals across all four scenarios. Meal types are indicated above each of the bars, scenario is indicated next to the corresponding point. Underlying data for this figure are available in the corresponding tab in Supporting Information S2 Excel file.

On a per serving basis, there initially appears to be a trade‐off between nutrition and environmental impact—the plant‐forward meals in each country have both a lower climate and biodiversity impact and a lower NRF15.3 score (Figure 5). However, the delta in NRF score in Germany is down to a single commonly supplemented nutrient, and the limiting amino acid (lysine) is commonly found in other elements of the diet (Matthews, 2020). In Indonesia, there were no limiting amino acids in either meal, and the foods driving the difference in NRF score in Indonesia (beans and banana) are minor contributors to the overall environmental impact of the meal, suggesting that nutritional quality is not causally correlated to environmental impact, and that it is possible to make substitutions that maintain or decrease environmental impact while increasing nutritional quality.

## 4 DISCUSSION

In this study we applied the Futura scenario framework to evaluate expected future environmental impacts of four meal options in two distinct cultural settings. While there are various modes of LCA that aim to assess the environmental performance of future systems, the methodological approach we have used aligns with the framing of pLCA outlined by Guinée et al. (2018) and Arvidsson et al. (2024). We observe that pLCA can be implemented in at least two distinct ways, that is, via the life cycle inventory (LCI) or the life cycle impact assessment method (LCIA). Our approach focused on adapting current LCIs to better reflect anticipated future technologies, like the methods employed by Beltran et al. (2020), Gibon et al. (2015), Hulst et al. (2020), Spielmann et al. (2005), and Walser et al. (2011). Whilst our case study was limited to four meals, we demonstrate the utility of the approach and observe that the database‐wide implementation of future SSP scenarios presented here could be extended to all agricultural products. We note that it is also possible to conduct pLCAs by adapting LCIA methods to reflect anticipated future conditions. For example, Baustert et al. (2022) have developed prospective characterization factors for water scarcity, which they have applied to a case study on water desalination for the steel industry. Such future water scarcity effects may also be relevant for agriculture, although we have not attempted to apply these characterization factors in our work, given our focus on climate footprints and land‐based biodiversity footprints.

Our findings, on a per serving basis for the meals, reinforce the general observation that reducing meat consumption is correlated with lower environmental impacts. The plant‐forward meal variants in both Germany and Indonesia had a lower climate and biodiversity impact than their meat‐based equivalents, per serving, under all present and future scenarios. Interestingly, however, the magnitude of the environmental benefit seen as a result of switching from a meat‐based to a plant‐forward meal differed markedly between the two food cultures, as represented by the meals studied which are known to be popular and widely consumed within the chosen countries (Figure 1). The reduction in impact achieved for the German plant‐forward meal example was of far greater consequence than for the Indonesian plant‐forward meal example due to the Indonesian plant‐forward meal still containing some animal‐based ingredients (consistent with a flexitarian diet).

Insights from our study suggest that in developed nations such as Germany, where nutrient security is all but assured, demand‐side actions, driven by meal choices, are both possible and effective, and should remain a priority for action to reduce the overall environmental impact of the global food system. For developing countries, where nutrient security is more at risk, and where food choices are more likely to be constrained by cost and availability, both the feasibility and the environmental effectiveness of switching from meat‐based to plant‐forward meals could be less certain. Indeed, when considered on an energetic basis (impact per 100 kcal) the less energy‐dense plant‐forward meal that we studied had a higher environmental impact for both biodiversity and climate impacts than the meat‐based meal. Thus, while “plant‐forward = good for the planet” may be a useful heuristic for western meals and diets, the situation is likely to be more nuanced in other food cultures, particularly where lower impact animal products are commonplace and where the alternative plant‐forward meal still contains animal‐based ingredients.

The prospective analysis presented here demonstrates the complementary importance of supply‐side measures. Over time and across scenarios, the climate and biodiversity footprints of the meals generally declined and showed similar trends in scenario responses: greatest footprint reductions for SSP1, that is, the most ambitious scenario with regard to sustainability; moderate reductions for the business‐as‐usual SSP2 scenario; and smallest reductions for the SSP3 scenario results, that is, a scenario assuming increases in animal‐based consumption and low levels of agricultural efficiency improvements. However, the magnitude of the change in impact from the baseline to the future scenarios for a given meal differed substantially. The effect of scenario change for the meat‐based meals was greater than for the plant‐forward meals, particularly for the sustainable development focused SSP1 scenario. These findings reflect the fact that actions taken to reduce climate and biodiversity impacts under the SSP scenarios are heterogeneous, both in their application and their effectiveness.

In line with findings in the literature (Poore & Nemecek, 2018), our results indicate that climate footprints of the meals are largely determined by activities up to farm gate. Consequently, changes modeled in the future scenarios that related to agricultural efficiency and practices were the main drivers of impact reduction. For meat and meat‐based meals, we found that feed inputs (grass and fodder crops) and livestock systems (feed conversion efficiency and feed composition) account for a large portion of the overall impact, which is in accordance with findings from others (Alig et al., 2012; Jungbluth, 2000; Nemecek et al., 2016). While similar drivers of change were observed for plant‐based ingredient cultivation as for feed, the effect of these agricultural efficiencies on climate and biodiversity indicators was lower. Even under the most ambition scenario (SSP1) the impacts of the meat‐based meals were still higher on a per serving basis than their plant‐forward equivalents. Thus, while supply‐side measures cannot compensate for demand‐side meal switching in terms of climate mitigation, they are a complementary and important part of ambitious, hotspot focused pathways for sustainable development. Indeed, such supply‐side measures are likely to be vital in overcoming the social, cultural, and nutritional trade‐offs inherent in dietary shifts.

In this study, while the meals substitutions considered were culturally equivalent (and therefore representative of a “real world” consumer decision), they were not nutritionally equivalent. Both plant‐forward options had lower overall NRF15.3 scores as well as lower environmental impacts per serving; this suggests a trade‐off between nutrition and the environment. However, meals are never eaten in isolation—they form part of a wider pattern of food consumption over a day, year, or lifetime—and as a consequence these results cannot be viewed in isolation either. Our nutritional assessment for the German meals suggests that Vitamin B12 is the key driver of difference in the NRF index between the meat‐based and plant‐forward meals. Vitamin B12 is rarely found naturally in plant‐based food items and is usually obtained from animal‐derived products, fortified products, or dietary supplements (Watanabe et al., 2014). It is acknowledged that Vitamin B12 availability may become critical when moving to more plant‐based diets (Willet et al., 2019). This suggests that the true trade‐off is not between the nutritional value and environmental impact of the meal itself, but between the meal‐switching decision and the implied subsequent decision about supplementation. Supplementation of such nutrients may be a key component of a societal shift to lower impact meal alternatives while ensuring continued nutritional quality. For the Indonesian case study, the difference in calorie content between the two meal options considerably affected the NRF and environmental impact results per 100 kcal. In a region where food security and undernourishment are ongoing concerns (World Food Programme, 2023), the calorie content of a meal is likely to be a key consideration. The western paradigm of low‐calorie alternatives being something to strive for is effectively reversed (at least for a substantial subsection of the population). This demonstrates a challenge with applying an energetic functional unit when comparing meal options in this context.

## 5 CONCLUSION

The macro‐level transitions required to minimize climate change and biodiversity loss are going to be made up of innumerable micro‐transitions enacted by individuals. This is why taking a bottom‐up perspective, focusing on potential real‐world changes in meal habits within different cultures provides a valuable insight into the transition to a lower impact food system. Familiarity, enjoyability, nutritional viability, and cost are vital considerations in the transition to sustainable food systems. Our analysis supports the need for complementary social and nutritional assessments to ensure socio‐cultural differences are considered when evaluating environmental impacts of foods (Green et al., 2020; McAuliffe et al., 2020). That said, our analysis also demonstrates that demand‐side transitions from meat to more plant‐based eating will be critical for climate mitigation and for bending the curve on biodiversity loss. In some settings, for instance where food and nutritional security are not guaranteed, these transitions may be partial and will need to be supported with supply‐side measures to improve production efficiency. Further work is required to consider the potential environmental impacts of “leakage” effects, for instance where missing nutrients are compensated via supplementation. Such impacts should be assessed via the type of prospective analysis demonstrated in this study.

## CONFLICT OF INTEREST STATEMENT

The authors declare no conflict of interest.

## Supporting information


**Supporting information S1**: This supporting information contains detailed information regarding the implementation of the SSPs in the prospective LCA (section 1), links to the supplementary datasets (section 2), loss factors for the Biodiversity Intactness Index (Table S1), recipe information and key life cycle inventory data (Tables S2 – S6) and details of the calculation of nutritional indicators for each of the meals (section 7).

**Supporting information S2**: This supporting information provides tabular numerical data which was used to generate Figures 2, 3, 4 and 5 in the manuscript.

## Data Availability

Data available on request from the authors.
