# Supplementary material for: Prospective life cycle assessment of climate and biodiversity impacts of meat‐based and plant‐forward meals: A case study of Indonesian and German meal options
Source: J Ind Ecol. 2024 Aug 16;28(6):1598–611. doi: 10.1111/jiec.13549 (PMC11667662; doi:10.1111/jiec.13549)
Supplement: Supplementary file 1 — Supporting information S1: This supporting information contains detailed information regarding the implementation of the SSPs in the prospective LCA (section 1), links to the supplementary datasets (section 2), loss factors for the Biodiversity Intactness Index (Table S1), recipe information and key life cycle inventory data (Tables S2 – S6) and details of the calculation of nutritional indicators for each of the meals (section 7). [file JIEC-28-1598-s001.docx]

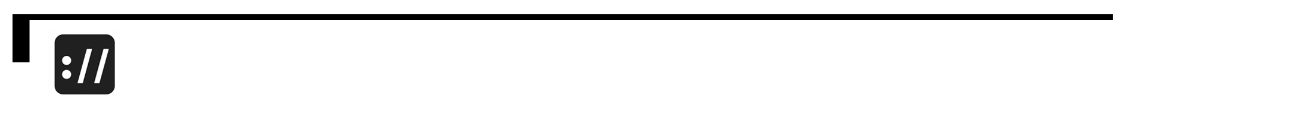


SUPPORTING INFORMATION FOR:

Marquardt, S. G., Joyce, P. J., Rigarlsford, G., Dötsch-Klerk, M., van Elk, K., Doelman, J., Daioglou, V., Huijbregts, M. A. J, Sim, S. Prospective Life Cycle Assessment of climate and biodiversity impacts of meat-based and plant-forward meals: A case study of Indonesian and German meal options. *Journal of Industrial Ecology.*


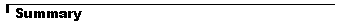
This supporting information contains detailed information regarding the implementation of the SSPs in the prospective LCA (section 1), links to the supplementary datasets (section 2), loss factors for the Biodiversity Intactness Index (Table S1), recipe information and key life cycle inventory data (Tables S2 – S6) and details of the calculation of nutritional indicators for each of the meals (section 7).


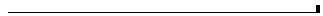


# Prospective LCA – filtering of relevant database entries

To implement projected changes from the Shared Socio-economic Pathway (SSP) scenarios from IMAGE for relevant activities in the LCI background system, we needed to systematically identify relevant activity datasets and map them to the relevant IMAGE identifiers. Both, ecoinvent (Wernet et al., 2016) and the WFLDB (Nemecek et al., 2019), employ a hierarchical naming convention for their activity datasets with ecoinvent also distinguishing between ‘transformation’ and ‘market’ activities. Ecoinvent entries are classified based on ‘special activity type’ (transformation or market), ‘product name’ and ‘activity name’. The WFLDB generally distinguishes between ‘category’ and ‘name’. For our purposes, we split the ‘category’ identifier into ‘product category’, ‘product name’, ‘product subgroup’ and ‘feed ingredients’ (if relevant).

For electricity technology and grid mix updates, we first isolated all activities for which ‘product name’ contained the string ‘electricity’ and the unit was ‘kWh’. From this list, we narrowed down the number of entries to entries linked to ‘electricity, low voltage’, ‘electricity, medium voltage’, ‘electricity, high voltage’. All entries classified as ‘market activity’ were considered for the electricity grid mix update. For the technical efficiency update, entries classified as ‘transformation’ activity were mapped to IMAGE technologies using the mapping provided by Beltran et al. (2020) as a starting point and including further adjustments due to differences in ecoinvent version (they used ecoinvent 3.3) and the introduction of additional technologies in IMAGE. In a second step, we needed to isolate the relevant energy carriers that would be affected by changes in efficiency (i.e., energy carriers used as ‘inputs from technosphere’ in electricity generation datasets). Filtering was done based on activity entries containing certain identifiers (hard coal, lignite, heavy fuel oil, wood chip, wood pellet, natural gas, biogas) in their ‘product name’ in combination with specific entries for unit (kg or m^3^) and ‘special activity type’ being ‘market activity’ or ‘market group’.

Crop entries from both ecoinvent and the WFLDB were considered for the filtering of relevant activity datasets for food and feed production. Isolating relevant ecoinvent entries required the use of an additional identifier, the ISIC (International Standard of Industrial Classification) class. We narrowed down the list of relevant entries by first filtering all entries for which the ISIC class description contained the string ‘Growing’. Using the additional information on the CPC (Central Product Classification) classification, we were able to establish a link to the FAO crop classification (i.e., the underlying classification for IMAGE crops). WFLDB entries were filtered based on the ‘product category’ being ‘plant products’ and the ‘product subcategory’ excluding ‘Grass for fodder and pasture’. Using the ‘product name’ of the remaining entries, we established a mapping to the FAO crop classification. To distinguish between irrigated and rain-fed crop activities, we mapped all WFLDB entries to irrigated IMAGE crops if the ‘activity name’ contained the string ‘irrigated’. See Supplementary data for mapping.

For livestock updates related to feed conversion efficiency and changes in feed basket composition, we considered entries form the WFLDB. Isolating relevant entries was based on ‘product name’ and ‘product category’ being ‘Animal products’, ‘product subcategory’ being ‘at farm’, ‘product subgroup’ being ‘Archetypes’ and unit ‘kg’. Poultry datasets have a different structure without the ‘product subgroup’ specification and were thus filtered excluding this specification. Mapping to IMAGE management systems was based on the ‘activity name’ containing ‘feedlot or intensive’ or ‘industrial’ for intensive systems and ‘grassland system’ of ‘backyard’ for extensive systems. For each livestock entry relevant ‘inputs from technosphere’ needed to be identified for the feed efficiency update. Relevant inputs were considered for the update if they related to:

- Feed intake (product subcategory: Animal production, product name: Feed, product subgroup: Feed baskets)
- Manure management (product subcategory: Animal production, product name: Manure management)
- Enteric emissions (product subcategory: Animal production, product name: Specific emissions, product name: contains ‘enteric’)
- Feed storage and transport
  - Non-dairy livestock (product subcategory: Animal production, activity name: contains ‘Feed storage and transport’)
  - Dairy livestock (product subcategory: Plant production, product name: installations, activity name: contains ‘storage’ or ‘silo’, unit: m3

Relevant entries for feed basked composition were isolated by filtering for ‘product subcategory’ being ‘Animal production’, ‘product name’ being ‘Feed’ and ‘product subgroup’ being ‘Feed mixtures’. For dairy cattle, the WFLDB does not implement feed intake via feed baskets but directly includes the feed ingredients in the dairy cattle datasets. To alter feed composition for dairy cattle, we needed to isolate relevant feed ingredients directly via ‘product subcategory’ being ‘Animal production’, ‘product name’ being ‘Feed’ and ‘product subgroup’ being ‘Feed ingredients’. For ‘Grazed grass’ as feed ingredient, further specification of the ‘activity name’ containing ‘country mix’, ‘global mix’ or ‘regional mix’ was necessary and for ‘Hay’ a specification of ‘activity name’ containing ‘production mix’. See Supplementary data for mapping.

# Supplementary data

The supplementary data can be accessed from <https://github.com/SandraMarquardt/PlantBasedMealsPublication.git>

# Biodiversity Intactness Index (BII) loss factors

**Table S1:** Biodiversity Intactness Index (BII) loss factors per land use type

| **Ecoinvent 3.5 land use type** | **Classification in Newbold et al., 2016** | **Biodiversity Intactness Index (BII) loss factors used in this paper** |
| --- | --- | --- |
| Occupation, agricultural peatland | Cropland - intense | 0.35 |
| Occupation, agriculture | Cropland - intense | 0.35 |
| Occupation, agriculture, mosaic | Cropland - intense | 0.35 |
| Occupation, annual crop | Cropland - light | 0.36 |
| Occupation, annual crop, conservation tillage | Cropland - light | 0.36 |
| Occupation, annual crop, conventional tillage | Cropland - intense | 0.35 |
| Occupation, annual crop, flooded crop | Cropland - intense | 0.35 |
| Occupation, annual crop, greenhouse | Cropland - intense | 0.35 |
| Occupation, annual crop, integrated | Cropland - light | 0.36 |
| Occupation, annual crop, irrigated | Cropland - intense | 0.35 |
| Occupation, annual crop, irrigated, extensive | Cropland - intense | 0.35 |
| Occupation, annual crop, irrigated, intensive | Cropland - intense | 0.35 |
| Occupation, annual crop, non-irrigated | Cropland - light | 0.36 |
| Occupation, annual crop, non-irrigated, diverse-intensive | Cropland - light | 0.36 |
| Occupation, annual crop, non-irrigated, extensive | Cropland - light | 0.36 |
| Occupation, annual crop, non-irrigated, fallow | Cropland - light | 0.36 |
| Occupation, annual crop, non-irrigated, intensive | Cropland - light | 0.36 |
| Occupation, annual crop, non-irrigated, monotone-intensive | Cropland - light | 0.36 |
| Occupation, annual crop, organic | Cropland - light | 0.36 |
| Occupation, annual crop, reduced tillage | Cropland - light | 0.36 |
| Occupation, arable land, unspecified use | Cropland - light | 0.36 |
| Occupation, construction site | Urban - intense | 0.29 |
| Occupation, cropland fallow (non-use) | Cropland - light | 0.36 |
| Occupation, dump site | Urban - intense | 0.29 |
| Occupation, dump site, benthos | Urban - intense | 0.29 |
| Occupation, forest, extensive | Secondary vegetation - light | 0.14 |
| Occupation, forest, intensive | Secondary vegetation - light | 0.14 |
| Occupation, forest, intensive, clear-cutting | Secondary vegetation - light | 0.14 |
| Occupation, forest, intensive, normal | Secondary vegetation - light | 0.14 |
| Occupation, forest, intensive, short-cycle | Secondary vegetation - light | 0.14 |
| Occupation, forest, natural | Primary vegetation - minimal | 0.00 |
| Occupation, forest, primary (non-use) | Primary vegetation - minimal | 0.00 |
| Occupation, forest, secondary (non-use) | Secondary vegetation - minimal | 0.06 |
| Occupation, forest, unspecified | Secondary vegetation - light | 0.14 |
| Occupation, forest, used | Secondary vegetation - light | 0.14 |
| Occupation, grassland | Pasture - minimal | 0.09 |
| Occupation, grassland, natural (non-use) | Primary vegetation - minimal | 0.00 |
| Occupation, grassland, natural, for livestock grazing | Pasture - minimal | 0.09 |
| Occupation, grassland/pasture/meadow | Pasture - minimal | 0.09 |
| Occupation, pasture, man-made | Pasture - intense | 0.45 |
| Occupation, pasture, man-made, extensive | Pasture - light | 0.31 |
| Occupation, pasture, man-made, intensive | Pasture - intense | 0.45 |
| Occupation, pasture, man-made, organic | Pasture - light | 0.31 |
| Occupation, permanent crop | Cropland - light | 0.36 |
| Occupation, permanent crop, fruit | Cropland - light | 0.36 |
| Occupation, permanent crop, fruit, extensive | Cropland - light | 0.36 |
| Occupation, permanent crop, fruit, intensive | Cropland - intense | 0.35 |
| Occupation, permanent crop, irrigated | Cropland - intense | 0.35 |
| Occupation, permanent crop, irrigated, extensive | Cropland - intense | 0.35 |
| Occupation, permanent crop, irrigated, intensive | Cropland - intense | 0.35 |
| Occupation, permanent crop, non-irrigated | Cropland - light | 0.36 |
| Occupation, permanent crop, non-irrigated, extensive | Cropland - light | 0.36 |
| Occupation, permanent crop, non-irrigated, intensive | Cropland - light | 0.36 |
| Occupation, permanent crop, vine | Cropland - light | 0.36 |
| Occupation, permanent crop, vine, extensive | Cropland - light | 0.36 |
| Occupation, permanent crop, vine, intensive | Cropland - intense | 0.35 |
| Occupation, urban | Urban - intense | 0.29 |
| Occupation, urban, continuously built | Urban - intense | 0.29 |
| Occupation, urban, discontinuously built | Urban - intense | 0.29 |
| Occupation, urban, green areas | Urban - intense | 0.29 |
| Occupation, urban/industrial fallow (non-use) | Urban - intense | 0.29 |

**Note:** BII loss factors were derived from the abundance factors in Figures S2 from Newbold et al. (2016) and corrected for compositional similarity (see Supplementary Material in Newbold et al., 2016 for a detailed description of the approach).

# Lifecycle phases and key assumptions

**Table S2:** LCA phases and key assumptions

| **LCA phase** | **Activity** | **Assumption, background** | **Data sources** |
| --- | --- | --- | --- |
| Agriculture | Production | Assumed to be region- and ingredient-specific to reflect international sourcing of raw materials. We used proxy region and ingredient information in cases of data gaps (e.g., for ingredients that are likely sourced from Indonesia, we assumed similar production as in China and/or India). Direct emissions were included for N_2_O (IPCC Tier1), CO_2_ (IPCC Tier 1), NO_x_ (EMEP/EEA Tier 1) and NH_3_ (EMEP/EEA Tier 1) | FAOSTAT (2020a, 2020b, 2020c) (relevant source regions for domestic supply)  WFLDB 3.5 (Nemeck et al., 2019) (for agricultural production^1^)  IPCC (2006)  EMEP/ EEA (2019) |
| Processing | Transformation  Transport | Assumed to take place domestically using domestically and internationally sourced raw materials.  Default domestic transport: 200km (WFLDB 3.5 default assumption)  International transport distances: Bee-line between major ports and/or borders using GoogleMaps (approach based on Eberle and Fels, 2016) | WFLDB 3.5 (Nemeck et al., 2019) (for agricultural production^1^)  Google (n.d.) |
|  | Packaging | If excluded in the WFLDB 3.5 datasets, we generated additional datasets based on Agribalyse (choice of packaging material and production of the packaging material). Inputs used in these datasets were adapted to the case study regions. In accordance with Agribalyse, we excluded secondary and tertiary packaging. | WFLDB 3.5 (Nemeck et al., 2019) (for agricultural production^1^)  Agribalyse (Asselin-Balençon et al., 2020) |
| Distribution and retail | Transport | Domestic transport of ingredients from farm or processing plant using WFLDB 3.5 default assumption (200 km) | WFLDB 3.5 (Nemeck et al., 2019) (for agricultural production^1^) |
|  | Storage | All fresh agricultural produce were assumed to have a cold-chain requiring chilled storage at distribution and retail. Chilled and ambient storage (energy requirements) were modeled based on Agribalyse. Data sets were adapted to the case study regions. | Agribalyse (Asselin-Balençon et al., 2020) |
| Consumer use | Storage | Based on Agribalyse, ambient storage was assumed for fresh agricultural produce. Data sets were adapted to the case study regions. | Agribalyse (Asselin-Balençon et al., 2020) |
|  | Preparation | Based on recipe specifications and using WFLDB 3.5 datasets. Data sets were adapted to the case study region. | WFLDB 3.5 (Nemeck et al., 2019) (for agricultural production^1^) |

**Note:** If the relevant product was not included in the WFLDB, we used the following data hierarchy to generate an appropriate dataset: ecoinvent 3.5 cut-off unit process (Wernet et al., 2016), Poore and Nemecek (2018), Agribalyse v3.0 (Asselin-Balençon et al., 2020) additional literature.

# Case study: Germany

**Table S3:** Recipe specification for the German case study meals

|  |  | **Meat-based meal**  **‘Spaghetti Bolognese with beef’** | **Plant-based meal**  **‘Spaghetti Bolognese with lentils’** |
| --- | --- | --- | --- |
| Ingredients | Tomatoes | 300 g | 300 g |
|  | Pasta | 250 g | 250 g |
|  | Beef mince | 200 g |  |
|  | Canned Lentils |  | 265 g |
|  | Water | 100 g | 100 g |
|  | Knorr mealmaker | 1 sachet (43 g) | 1 sachet (43 g) |
|  | Olive oil | 15 g | 15 g |
| Number of servings |  | 3 | 3 |
| Meal preparation |  | - - Fry beef/ lentils in oil   - Add tomatoes, water and mealmaker and let simmer for 10 minutes   - Boil pasta in 1 liter of water | |

**Table S4:** Life Cycle Inventory key assumption

| **Ingredient** | **Packaging** | **Storage** | **Key database reference** | **Data sources** |
| --- | --- | --- | --- | --- |
| Tomatoes | None | Distribution: chilled  Retail: chilled  Home: ambient | Tomato, fresh grade, at farm | WFLDB 3.5;  Agribalyse v3.0 |
| Pasta | LDPE | Distribution: ambient  Retail: ambient  Home: ambient | Pasta, dried, from durum wheat, at plant | WFLDB 3.5; Agribalyse v3.0 |
| Beef mince | LDPE | Distribution: chilled  Retail: chilled  Home: chilled | Beef, fresh meat, at slaughterhouse | WFLDB 3.5; Agribalyse v3.0 |
| Canned lentils | Can | Distribution: ambient  Retail: ambient  Home: ambient | Lentils, dry, at farm | WFLDB 3.5; Agribalyse v3.0 |
| Water |  |  | Tap water | ecoinvent 3.5 |
| Knorr mealmaker | Aluminum | Distribution: ambient  Retail: ambient  Home: ambient | None; own recipe | UL product specifications |
| Olive oil | PET | Distribution: ambient  Retail: ambient  Home: ambient | Olive oil, at oil mill | WFLDB 3.5; Agribalyse v3.0 |
| Boiling (pasta, meal) |  |  | Boiling, vegetables, with electric stove | WFLDB 3.5 |
| Frying (beef, lentils) |  |  | Frying, vegetables, with electric stove | WFLDB 3.5 |

**Note:** Data from the different data sources used for the life cycle phases of an ingredient were used according to the description in Section 4.2.2 (‘Baseline LCA’) and as document in Table A.4.2.

# Case study: Indonesia

**Table S5**: Recipe specification for the Indonesian case study meals

|  |  | **Meat-based meal**  **‘Chicken soup, fried beans, rice, fruit** | **Plant-based meal**  **‘Tofu soup, omelet, rice, fruit’** |
| --- | --- | --- | --- |
| Soup | Ingredients | 300 g chicken meat | 200 g carrots |
|  |  | 150 g potatoes | 150 g tofu |
|  |  | 150 g carrots | 50 g moringa leaves |
|  |  | 50 g cabbage | 50 g sweet corn |
|  |  | 50 g moringa leaves | 12 g shallots |
|  |  | 30 g spring beans | 6 g Royco chicken seasoning |
|  |  | 24 g onions | 1.3 g nutmeg* |
|  |  | 20 g shallots | 1 g sugar* |
|  |  | 20 g garlic | 500 g water |
|  |  | 20 g palm oil |  |
|  |  | 10 g celery |  |
|  |  | 10 g Royco chicken seasoning |  |
|  |  | 0.8 g nutmeg* |  |
|  |  | 1 liter water |  |
|  | Number of servings | 4 | 4 |
|  | Meal preparation | - - In a pan/wok sauté shallots and garlic until translucent   - Remove and set aside   - In saucepan bring water to boil and add sautéed onions   - Add chicken and cook until half-done   - Add potatoes and carrots. Cook until broth is reduced to 750ml   - Add cabbage, beans, moringa leaves, onion, celery, Royco seasoning and nutmeg and bring to boil | - - Bring water to boil   - Add onions and carrots and cook for a few minutes   - Add corn and tofu and cook until done   - Add moringa, Royco seasoning, sugar and nutmeg and bring to boil |
| Side dish (fried beans / omelet) | Ingredients | 350 g spring beans | 275 g egg |
|  |  | 25 g shallots | 100 g cabbage |
|  |  | 20 g garlic | 50 g chicken meat |
|  |  | 20 g Bango kecap manis | 50 g soybean sprouts |
|  |  | 10 g palm oil | 20 g palm oil |
|  |  | 8 g chillis | 10 g onions |
|  |  | 4.5 g Royco chicken seasoning | 9 g soy sauce |
|  |  | 2 g ginger* | 6 g Royco chicken seasoning |
|  |  | 0.4 g bay leaf* | 3 g pepper* |
|  |  | 0.1 g pepper* |  |
|  |  | 100 g water |  |
|  | Number of servings | 4 | 6 |
|  | Meal preparation | - - Heat oil in pan/wok over medium heat   - Sauté shallots and garlic until translucent   - Add chilli, ginger and bay leaves and sauté for 2-3 minutes   - Add beans, Bango kecap manis, Royco seasoning, pepper and water   - Continue cooking until beans are cooked and liquid is reduced | - - Mix all ingredients in a bowl (except oil)   - Heat oil in non-sticking pan and cook egg-mix until well done   - Remove from heat and cut into desired portions |
| Rice | Ingredients | 150 g | 150 g |
|  | Number of servings | 1 | 1 |
|  | Meal preparation |  |  |
| Fruit | Ingredients | 50 g Banana | 270 g Watermelon |
|  | Number of servings | 1 | 1 |
|  | Meal preparation |  |  |

**Note:** *Omitted in recipe implementation. Only ingredients contributing more than 1% (weight-based contribution) were considered in the implementation.

**Table S6:** Life Cycle Inventory key assumption

| **Ingredient** | **Packaging** | **Storage** | **Key database reference** | **Data sources** |
| --- | --- | --- | --- | --- |
| Chicken meat | LDPE | Distribution: chilled  Retail: chilled  Home: ambient | Chicken, fresh meat, at slaughterhouse | WFLDB 3.5;  Agribalyse v3.0 |
| Carrots | None | Distribution: chilled  Retail: ambient  Home: ambient | Carrot, at farm | WFLDB 3.5;  Agribalyse v3.0 |
| Potatoes | None | Distribution: chilled  Retail: ambient  Home: ambient | Potato, at farm | WFLDB 3.5;  Agribalyse v3.0 |
| Cabbage | None | Distribution: chilled  Retail: ambient  Home: ambient |  | Poore and Nemecek (2018);  Agribalyse v3.0 |
| Moringa leaves | None | Distribution: chilled  Retail: ambient  Home: ambient |  | Biswas (2008);  Radovich (2011);  Agribalyse v3.0 |
| Beans | None | Distribution: chilled  Retail: ambient  Home: ambient |  | Poore and Nemecek (2018);  Agribalyse v3.0 |
| Onions, shallots | None | Distribution: chilled  Retail: ambient  Home: ambient | Onion, at farm | WFLDB 3.5;  Agribalyse v3.0 |
| Garlic | None | Distribution: chilled  Retail: ambient  Home: ambient | Garlic, at farm | WFLDB 3.5;  Agribalyse v3.0 |
| Palm oil | PET | Distribution: ambient  Retail: ambient  Home: ambient | Palm oil, crude, at oil mill | WFLDB 3.5;  Agribalyse v3.0 |
| Celery | None | Distribution: chilled  Retail: ambient  Home: ambient | Celery | ecoinvent 3.5;  Agribalyse v3.0 |
| Water |  |  | Tap water | ecoinvent 3.5 |
| Royco chicken | Aluminium | Distribution: ambient  Retail: ambient  Home: ambient |  | UL product specification |
| Chilli | None | Distribution: chilled  Retail: ambient  Home: ambient | Chilli, fresh, at farm | WFLDB 3.5;  Agribalyse v3.0 |
| Bango kecap manis | LDPE | Distribution: chilled  Retail: chilled  Home: chilled |  | UL product specification |
| Rice | LDPE | Distribution: ambient  Retail: ambient  Home: ambient | Rice, at farm | WFLDB 3.5;  Agribalyse v3.0 |
| Banana | None | Distribution: chilled  Retail: ambient  Home: ambient | Banana, at farm | WFLDB 3.5;  Agribalyse v3.0 |
| Tofu | PP | Distribution: chilled  Retail: chilled  Home: chilled | Tofu, at plant | ecoinvent 3.5;  WFLDB 3.5;  Agribalyse v3.0 |
| Sweet corn | None | Distribution: chilled  Retail: ambient  Home: ambient | Sweet corn, at farm | WFLDB 3.5;  Agribalyse v3.0 |
| Eggs | Cardboard | Distribution: ambient  Retail: ambient  Home: ambient | Chicken egg, poultry industrial laying systems, at farm | WFLDB 3.5;  Agribalyse v3.0 |
| Soybean sprouts | LDPE | Distribution: chilled  Retail: ambient  Home: ambient | Soybean, at farm | WFLDB 3.5;  Agribalyse v3.0 |
| Soy sauce | LDPE | Distribution: chilled  Retail: chilled  Home: chilled | Soy sauce | Agribalyse v3.0;  WFLDB 3.5 |
| Melon | None | Distribution: chilled  Retail: ambient  Home: ambient | Melon | ecoinvent 3.5  Agribalyse v3.0 |
| Boiling (chicken, chicken soup, beans, rice, tofu soup) |  |  | Boiling, vegetables, LPG stove | WFLDB 3.5 |
| Sauté (vegetables) |  |  | Boiling, vegetables, LPG stove | WFLDB 3.5 |
| Frying (omelet) |  |  | Frying, vegetables, LPG stove | WFLDB 3.5 |

# Nutritional values and scores

In order to calculate the relevant NRF15.3 index for each of the functional units outlined above, qualifying and disqualifying nutrient quantities per serving and per 100 kCal were benchmarked against the respective daily nutrient reference values from CODEX NRV and, for sugar, EU reference intake (European Parliament, 2011; Lewis, 2019). When a meal delivered 100% or more of the daily nutrient reference value, it was capped at 100%, to avoid overvaluing foods that provide very large amounts of a single nutrient. The final NRF score was calculated by subtracting the sum of disqualifying nutrients from the sum of qualifying nutrients, giving a maximum possible score of 1200 and a minimum possible score of -300. Each of the meals considered has an NRF15.3 score greater than 200. We used the NRF15.3 scores of the meals to put the results of our environmental assessment into a nutritional context.

## Nutrient profile and scores – German case studies

**Table S7:** Nutrient profile, NRF 15.3 and IAA scores per serving – German meal options

|  | **Meat-based meal 'Spaghetti Bolognese with beef'** | | |  | **Plant-based meal 'Spaghetti Bolognese with lentils'** | | |  |
| --- | --- | --- | --- | --- | --- | --- | --- | --- |
|  |  |  | % of recommended intake [capped at 100%] | |  |  | % of recommended intake [capped at 100%] | |
|  | Amount per serving | | per serving | per 100 kcal | Amount per serving | | per serving | per 100 kcal |
| **NRF 15.3** |  |  | 319 | 63 |  |  | 208 | 39 |
| **IAA** |  |  | 108 |  |  |  | 86 |  |
| Energy | 508.89 | kcal | 25% |  | 529.23 | kcal | 26% |  |
| Dietary Fibre | 7.32 | g | 29% | 6% | 11.43 | g | 46% | 9% |
| Protein | 25.66 | g | 51% | 10% | 21.41 | g | 43% | 8% |
| Histidine | 0.63 | g | 154% | 30% | 0.43 | g | 124% | 23% |
| Isoleucine | 1.07 | g | 139% | 27% | 0.84 | g | 131% | 25% |
| Leucine | 1.82 | g | 116% | 23% | 1.43 | g | 110% | 21% |
| Lysine L | 1.32 | g | 108% | 21% | 0.88 | g | 86% | 16% |
| SAA (Methonine + Cysteine) | 0.86 | g | 146% | 29% | 0.54 | g | 110% | 21% |
| AAA (Phenylalanine + Tyrosine) | 1.78 | g | 169% | 33% | 1.57 | g | 179% | 34% |
| Threonine | 0.89 | g | 139% | 27% | 0.68 | g | 127% | 24% |
| Tryptophan | 0.26 | g | 151% | 30% | 0.19 | g | 137% | 26% |
| Valine | 1.17 | g | 114% | 22% | 0.96 | g | 113% | 21% |
| Potassium | 591.05 | mg | 17% | 3% | 664.95 | mg | 19% | 4% |
| Calcium | 32.33 | mg | 3% | 1% | 65.88 | mg | 7% | 1% |
| Magnesium | 70.72 | mg | 23% | 4% | 104.87 | mg | 34% | 6% |
| Iron | 3.05 | mg | 22% | 4% | 4.23 | mg | 30% | 6% |
| Zinc | 4.66 | mg | 42% | 8% | 2.69 | mg | 24% | 5% |
| Folate DFE | 0.00 | µg | 0% | 0% | 0.00 | µg | 0% | 0% |
| Vitamin A | 108.85 | µg | 14% | 3% | 112.15 | µg | 14% | 3% |
| Vitamin D | 0.00 | µg | 0% | 0% | 0.00 | µg | 0% | 0% |
| Vitamin E | 1.87 | mg | 19% | 4% | 2.04 | mg | 20% | 4% |
| Vitamin B1 | 0.19 | mg | 16% | 3% | 0.19 | mg | 16% | 3% |
| Vitamin B2 | 0.21 | mg | 17% | 3% | 0.15 | mg | 12% | 2% |
| Vitamin B3 | 5.47 | mg | 36% | 7% | 2.65 | mg | 18% | 3% |
| Vitamin B12 | 3.20 | ug | 100%* | 20%* | 0.00 | ug | 0% | 0% |
| Vitamin C | 19.26 | mg | 19% | 4% | 19.34 | mg | 19% | 4% |
| Total Fats | 12.54 | g | 18% | 4% | 7.35 | g | 10% | 2% |
| Saturated Fat | 3.48 | g | 17% | 3% | 1.08 | g | 5% | 1% |
| Trans Fat | 0.00 | g | 0% | 0% | 0.00 | g | 0% | 0% |
| Monounsaturated Fat | 6.30 | g | 31% | 6% | 3.79 | g | 19% | 4% |
| Polyunsaturated Fat | 1.28 | g | 0% | 0% | 1.26 | g | 0% | 0% |
| Cholesterol | 38.05 | mg | 0% | 0% | 0.05 | mg | 0% | 0% |
| Sodium | 709.63 | mg | 35% | 7% | 915.46 | mg | 46% | 9% |
| Total Carbohydrates (excl. fibre) | 69.01 | g | 27% | 5% | 87.51 | g | 34% | 6% |
| Sugar | 8.29 | g | 9% | 2% | 9.22 | g | 10% | 2% |

**Note**:

*Capped value (i.e., no individual qualifying nutrient can exceed 100%)

Dark green/white font: NRF15.3 qualifying nutrients

Dark red/white font: NRF15.3 disqualifying nutrients

Blue/white font: indispensable amino acids (IAA) relevant for IAA score

## Nutrient profile and scores – Indonesian case studies

**Table S8:** Nutrient profile, NRF 15.3 and IAA scores per serving – Indonesian meal options

|  | **Meat-based meal 'Chicken soup, fried beans, rice, fruit'** | | |  | **Plant-based meal 'Tofu soup, omelet, rice, fruit'** | | |  |
| --- | --- | --- | --- | --- | --- | --- | --- | --- |
|  |  |  | % of recommended intake [capped at 100%] | |  |  | % of recommended intake [capped at 100%] | |
|  | Amount per serving | | per serving | per 100 kcal | Amount per serving | | per serving | per 100 kcal |
| **NRF 15.3** |  |  | 593 | 67 |  |  | 349 | 69 |
| **IAA** |  |  | 107 |  |  |  | 159 |  |
| Energy | 884.40 | kcal | 44% |  | 505.24 | kcal | 25% |  |
| Dietary Fibre | 28.52 | g | 100%* | 11% | 3.79 | g | 15% | 3% |
| Protein | 41.82 | g | 84% | 9% | 19.44 | g | 39% | 8% |
| Histidine | 1.34 | g | 200% | 23% | 0.72 | g | 231% | 46% |
| Isoleucine | 1.68 | g | 134% | 15% | 1.17 | g | 200% | 40% |
| Leucine | 3.06 | g | 120% | 14% | 2.08 | g | 175% | 35% |
| Lysine L | 3.30 | g | 164% | 19% | 1.82 | g | 195% | 39% |
| SAA (Methonine + Cysteine) | 1.39 | g | 144% | 16% | 0.71 | g | 159% | 31% |
| AAA (Phenylalanine + Tyrosine) | 2.80 | g | 163% | 18% | 2.05 | g | 258% | 51% |
| Threonine | 1.58 | g | 151% | 17% | 0.99 | g | 204% | 40% |
| Tryptophan | 0.40 | g | 145% | 16% | 0.29 | g | 223% | 44% |
| Valine | 1.80 | g | 107% | 12% | 1.35 | g | 173% | 34% |
| Potassium | 2098.99 | mg | 60% | 7% | 861.26 | mg | 25% | 5% |
| Calcium | 275.23 | mg | 28% | 3% | 253.91 | mg | 25% | 5% |
| Magnesium | 261.98 | mg | 85% | 10% | 95.48 | mg | 31% | 6% |
| Iron | 5.84 | mg | 42% | 5% | 5.20 | mg | 37% | 7% |
| Zinc | 3.71 | mg | 34% | 4% | 2.35 | mg | 21% | 4% |
| Folate DFE | 430.27 | µg | 100%* | 11% | 89.13 | µg | 22% | 4% |
| Vitamin A | 385.30 | µg | 48% | 5% | 652.91 | µg | 82% | 16% |
| Vitamin D | 0.30 | µg | 3% | 0% | 0.93 | µg | 9% | 2% |
| Vitamin E | 1.79 | mg | 18% | 2% | 1.52 | mg | 15% | 3% |
| Vitamin B1 | 0.74 | mg | 62% | 7% | 0.32 | mg | 27% | 5% |
| Vitamin B2 | 0.48 | mg | 40% | 5% | 0.47 | mg | 39% | 8% |
| Vitamin B3 | 11.94 | mg | 80% | 9% | 3.22 | mg | 21% | 4% |
| Vitamin B12 | 0.26 | ug | 11% | 1% | 0.43 | ug | 18% | 4% |
| Vitamin C | 37.54 | mg | 38% | 4% | 41.68 | mg | 42% | 8% |
| Total Fats | 17.51 | g | 25% | 3% | 12.83 | g | 18% | 4% |
| Saturated Fat | 6.25 | g | 31% | 4% | 4.05 | g | 20% | 4% |
| Trans Fat | 0.08 | g | 4% | 0% | 0.02 | g | 1% | 0% |
| Monounsaturated Fat | 5.92 | g | 30% | 3% | 4.26 | g | 21% | 4% |
| Polyunsaturated Fat | 3.55 | g | 0% | 0% | 3.16 | g | 0% | 0% |
| Cholesterol | 48.00 | mg | 0% | 0% | 173.92 | mg | 0% | 0% |
| Sodium | 1183.39 | mg | 59% | 7% | 882.60 | mg | 44% | 9% |
| Total Carbohydrates (excl. fibre) | 109.48 | g | 42% | 5% | 77.41 | g | 30% | 6% |
| Sugar | 13.07 | g | 15% | 2% | 21.51 | g | 24% | 5% |

Note:

*Capped value (i.e., no individual qualifying nutrient can exceed 100%)

Dark green/white font: NRF15.3 qualifying nutrients

Dark red/white font: NRF15.3 disqualifying nutrients

Blue/white font: indispensable amino acids (IAA) relevant for IAA score


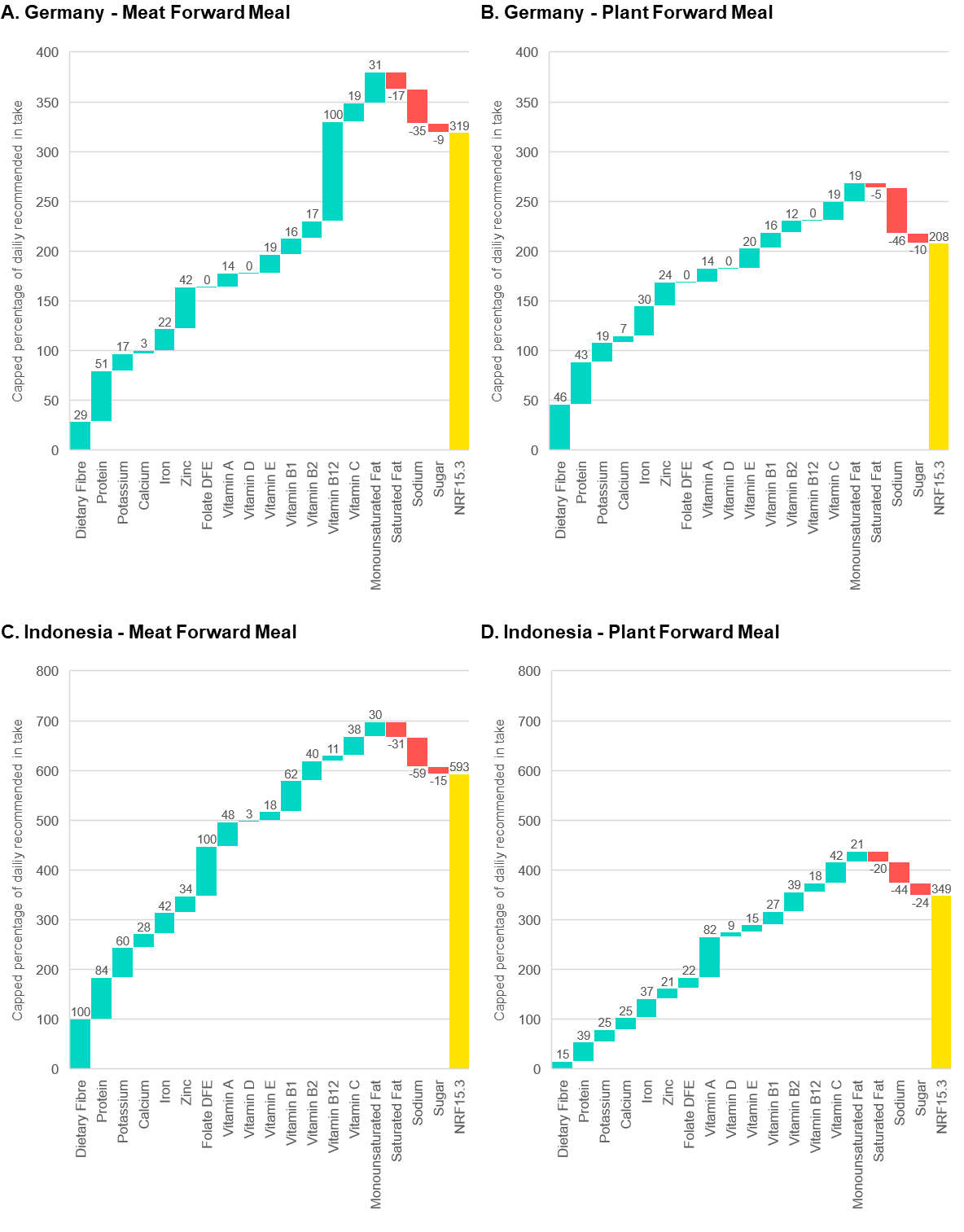
**Fig. S1.** NRF15.3 scores per serving and contributing nutrients for each of the four meals in this study

# Climate footprints per serving for meat-based and vegetarian meal options in Germany and Indonesia per clustered contributing processes

**Table S9:** Climate footprints per serving for meat-based and vegetarian meal options in Germany and Indonesia (2015 baseline, absolute scenario differences for 2050 and the scenarios SSP1, SSP2, SSP3 and inter-scenario variability benchmarked against the lowest 2050 scenario results) per clustered contributing processes

|  | **Contributing processes** | **Baseline** | **SSP1** | **SSP2** | **SSP3** | **SSP1** | **SSP2** | **SSP3** |
| --- | --- | --- | --- | --- | --- | --- | --- | --- |
|  |  | **2015** | **2050** | | | **2050** | | |
|  |  | [kg CO_2_eq per serving] | Absolute difference to baseline | | | inter-scenario variability (1=min 2050 scenario result) | | |
| **DE Meat-based meal 'Spaghetti Bolognese with beef'** | **Total** | **4.04** | **-0.72** | **-0.20** | **-0.12** | **1.00** | **1.16** | **1.18** |
|  | Plant production | 0.22 | -0.07 | -0.04 | -0.03 | 1.00 | 1.19 | 1.22 |
|  | Animal production | 2.31 | -0.29 | -0.04 | -0.01 | 1.00 | 1.12 | 1.14 |
|  | Fertilizers | 0.08 | -0.01 | 0.00 | 0.00 | 1.00 | 1.14 | 1.15 |
|  | Food products | 0.00 | 0.00 | 0.00 | 0.00 | 0.00 | 0.00 | 0.00 |
|  | Energy | 0.38 | -0.07 | 0.05 | 0.04 | 1.00 | 1.38 | 1.36 |
|  | Manufacturing | 0.08 | -0.01 | 0.00 | 0.00 | 1.00 | 1.07 | 1.09 |
|  | Industrial processes | 0.05 | -0.01 | 0.00 | 0.00 | 1.00 | 1.25 | 1.24 |
|  | Services | 0.11 | 0.00 | 0.00 | 0.00 | 1.00 | 1.03 | 1.02 |
|  | Other | 0.31 | -0.14 | -0.08 | -0.05 | 1.00 | 1.37 | 1.56 |
|  | Remaining components | 0.50 | -0.12 | -0.08 | -0.07 | 1.00 | 1.09 | 1.11 |
| **DE Plant-based meal 'Spaghetti Bolognese with lentils'** | **Total** | **0.70** | **-0.09** | **0.02** | **0.01** | **1.00** | **1.18** | **1.16** |
|  | Plant production | 0.05 | -0.01 | -0.01 | -0.01 | 1.02 | 1.00 | 1.01 |
|  | Animal production | 0.00 | 0.00 | 0.00 | 0.00 | 0.00 | 0.00 | 0.00 |
|  | Fertilizers | 0.01 | 0.00 | 0.00 | 0.00 | 1.02 | 1.00 | 1.02 |
|  | Food products | 0.00 | 0.00 | 0.00 | 0.00 | 1.00 | 1.00 | 1.00 |
|  | Energy | 0.37 | -0.06 | 0.03 | 0.03 | 1.00 | 1.29 | 1.27 |
|  | Manufacturing | 0.04 | 0.00 | 0.00 | 0.00 | 1.01 | 1.00 | 1.01 |
|  | Industrial processes | 0.04 | 0.00 | 0.00 | 0.00 | 1.00 | 1.13 | 1.10 |
|  | Services | 0.10 | 0.00 | 0.00 | 0.00 | 1.01 | 1.00 | 1.01 |
|  | Other | 0.00 | 0.00 | 0.00 | 0.00 | 1.00 | 1.07 | 1.04 |
|  | Remaining components | 0.10 | -0.02 | 0.00 | -0.01 | 1.00 | 1.19 | 1.11 |

|  | **Contributing processes** | **Baseline** | **SSP1** | **SSP2** | **SSP3** | **SSP1** | **SSP2** | **SSP3** |
| --- | --- | --- | --- | --- | --- | --- | --- | --- |
|  |  | **2015** | **2050** | | | **2050** | | |
|  |  | [kg CO_2_eq per serving] | Absolute difference to baseline | | | inter-scenario variability (1=min 2050 scenario result) | | |
| **ID Meat-based meal 'Chicken soup, fried beans, rice, fruit'** | **Total** | **1.61** | **-0.58** | **-0.51** | **-0.46** | **1.00** | **1.07** | **1.12** |
|  | Plant production | 0.24 | -0.06 | -0.05 | -0.05 | 1.00 | 1.01 | 1.04 |
|  | Animal production | 0.02 | 0.00 | 0.00 | 0.00 | 1.00 | 1.04 | 1.06 |
|  | Fertilizers | 0.01 | 0.00 | 0.00 | 0.00 | 1.01 | 1.00 | 1.16 |
|  | Food products | 0.01 | 0.00 | 0.00 | 0.00 | 1.00 | 1.01 | 1.02 |
|  | Energy | 0.51 | -0.27 | -0.27 | -0.26 | 1.00 | 1.03 | 1.05 |
|  | Manufacturing | 0.06 | 0.00 | 0.00 | 0.00 | 1.00 | 1.00 | 1.01 |
|  | Industrial processes | 0.03 | -0.01 | -0.01 | -0.01 | 1.00 | 1.02 | 1.04 |
|  | Services | 0.08 | -0.01 | -0.01 | 0.00 | 1.00 | 1.04 | 1.05 |
|  | Other | 0.48 | -0.18 | -0.13 | -0.09 | 1.00 | 1.15 | 1.30 |
|  | Remaining components | 0.18 | -0.05 | -0.05 | -0.05 | 1.00 | 1.05 | 1.04 |
| **ID Plant-based meal 'Tofu soup, omelet, rice, fruit'** | **Total** | **1.28** | **-0.43** | **-0.39** | **-0.36** | **1.00** | **1.05** | **1.08** |
|  | Plant production | 0.21 | -0.05 | -0.05 | -0.05 | 1.01 | 1.00 | 1.01 |
|  | Animal production | 0.10 | -0.01 | -0.01 | -0.01 | 1.00 | 1.04 | 1.06 |
|  | Fertilizers | 0.00 | 0.00 | 0.00 | 0.00 | 1.21 | 1.05 | 1.00 |
|  | Food products | 0.00 | 0.00 | 0.00 | 0.00 | 1.00 | 1.00 | 1.00 |
|  | Energy | 0.41 | -0.22 | -0.21 | -0.21 | 1.00 | 1.03 | 1.05 |
|  | Manufacturing | 0.05 | 0.00 | 0.00 | 0.00 | 1.02 | 1.00 | 1.00 |
|  | Industrial processes | 0.03 | -0.01 | -0.01 | -0.01 | 1.02 | 1.04 | 1.00 |
|  | Services | 0.08 | 0.00 | 0.00 | 0.00 | 1.00 | 1.00 | 1.01 |
|  | Other | 0.25 | -0.10 | -0.07 | -0.05 | 1.00 | 1.16 | 1.31 |
|  | Remaining components | 0.14 | -0.05 | -0.04 | -0.04 | 1.00 | 1.09 | 1.10 |

|  | **Contributing processes** | **Baseline** | **SSP1** | **SSP2** | **SSP3** | **SSP1** | **SSP2** | **SSP3** |
| --- | --- | --- | --- | --- | --- | --- | --- | --- |
|  |  | **2015** | **2050** | | | **2050** | | |
|  |  | [kg CO_2_eq per serving] | Absolute difference to baseline | | | inter-scenario variability (1=min 2050 scenario result) | | |
| **ID Meat-based meal 'Fried beans'** | **Total** | **0.21** | **-0.10** | **-0.10** | **-0.10** | **1.00** | **1.04** | **1.05** |
|  | Plant production | 0.01 | 0.00 | 0.00 | 0.00 | 1.00 | 1.01 | 1.02 |
|  | Animal production | 0.00 | 0.00 | 0.00 | 0.00 | 0.00 | 0.00 | 0.00 |
|  | Fertilizers | 0.01 | 0.00 | 0.00 | 0.00 | 1.02 | 1.00 | 1.00 |
|  | Food products | 0.00 | 0.00 | 0.00 | 0.00 | 1.00 | 1.01 | 1.01 |
|  | Energy | 0.13 | -0.09 | -0.09 | -0.09 | 1.00 | 1.08 | 1.09 |
|  | Manufacturing | 0.00 | 0.00 | 0.00 | 0.00 | 1.02 | 1.04 | 1.00 |
|  | Industrial processes | 0.01 | 0.00 | 0.00 | 0.00 | 1.03 | 1.04 | 1.00 |
|  | Services | 0.01 | 0.00 | 0.00 | 0.00 | 1.00 | 1.00 | 1.00 |
|  | Other | 0.02 | 0.00 | 0.00 | 0.00 | 1.00 | 1.05 | 1.08 |
|  | Remaining components | 0.02 | -0.01 | -0.01 | -0.01 | 1.00 | 1.06 | 1.05 |
| **ID Plant-based meal 'Omelet'** | **Total** | **0.56** | **-0.18** | **-0.14** | **-0.11** | **1.00** | **1.09** | **1.17** |
|  | Plant production | 0.04 | -0.01 | -0.01 | -0.01 | 1.00 | 1.06 | 1.13 |
|  | Animal production | 0.10 | -0.01 | -0.01 | -0.01 | 1.00 | 1.04 | 1.06 |
|  | Fertilizers | 0.00 | 0.00 | 0.00 | 0.00 | 1.98 | 1.03 | 1.00 |
|  | Food products | 0.00 | 0.00 | 0.00 | 0.00 | 1.58 | 1.00 | 1.04 |
|  | Energy | 0.07 | -0.03 | -0.03 | -0.03 | 1.00 | 1.01 | 1.01 |
|  | Manufacturing | 0.01 | 0.00 | 0.00 | 0.00 | 1.00 | 1.02 | 1.04 |
|  | Industrial processes | 0.01 | 0.00 | 0.00 | 0.00 | 1.00 | 1.05 | 1.00 |
|  | Services | 0.02 | 0.00 | 0.00 | 0.00 | 1.00 | 1.04 | 1.08 |
|  | Other | 0.25 | -0.10 | -0.07 | -0.05 | 1.00 | 1.16 | 1.31 |
|  | Remaining components | 0.06 | -0.02 | -0.02 | -0.01 | 1.00 | 1.12 | 1.21 |

|  | **Contributing processes** | **Baseline** | **SSP1** | **SSP2** | **SSP3** | **SSP1** | **SSP2** | **SSP3** |
| --- | --- | --- | --- | --- | --- | --- | --- | --- |
|  |  | **2015** | **2050** | | | **2050** | | |
|  |  | [kg CO_2_eq per serving] | Absolute difference to baseline | | | inter-scenario variability (1=min 2050 scenario result) | | |
| **ID Meat-based meal 'Chicken soup'** | **Total** | **1.02** | **-0.35** | **-0.29** | **-0.24** | **1.00** | **1.09** | **1.17** |
|  | Plant production | 0.09 | -0.02 | -0.02 | -0.02 | 1.00 | 1.04 | 1.09 |
|  | Animal production | 0.02 | 0.00 | 0.00 | 0.00 | 1.00 | 1.04 | 1.06 |
|  | Fertilizers | 0.00 | 0.00 | 0.00 | 0.00 | 1.09 | 1.02 | 1.00 |
|  | Food products | 0.00 | 0.00 | 0.00 | 0.00 | 1.00 | 1.02 | 1.03 |
|  | Energy | 0.24 | -0.11 | -0.10 | -0.10 | 1.00 | 1.03 | 1.03 |
|  | Manufacturing | 0.03 | 0.00 | 0.00 | 0.00 | 1.00 | 1.01 | 1.03 |
|  | Industrial processes | 0.02 | 0.00 | 0.00 | 0.00 | 1.02 | 1.00 | 1.03 |
|  | Services | 0.05 | 0.00 | 0.00 | 0.00 | 1.00 | 1.01 | 1.02 |
|  | Other | 0.46 | -0.17 | -0.13 | -0.08 | 1.00 | 1.16 | 1.31 |
|  | Remaining components | 0.11 | -0.04 | -0.03 | -0.03 | 1.00 | 1.13 | 1.20 |
| **ID Plant-based meal 'Tofu soup'** | **Total** | **0.18** | **-0.06** | **-0.06** | **-0.06** | **1.00** | **1.02** | **1.02** |
|  | Plant production | 0.01 | 0.00 | 0.00 | 0.00 | 1.03 | 1.00 | 1.03 |
|  | Animal production | 0.00 | 0.00 | 0.00 | 0.00 | 0.00 | 0.00 | 0.00 |
|  | Fertilizers | 0.00 | 0.00 | 0.00 | 0.00 | 1.00 | 1.05 | 1.13 |
|  | Food products | 0.00 | 0.00 | 0.00 | 0.00 | 1.00 | 1.00 | 1.00 |
|  | Energy | 0.11 | -0.05 | -0.05 | -0.05 | 1.00 | 1.03 | 1.03 |
|  | Manufacturing | 0.01 | 0.00 | 0.00 | 0.00 | 1.00 | 1.00 | 1.00 |
|  | Industrial processes | 0.01 | 0.00 | 0.00 | 0.00 | 1.00 | 1.01 | 1.03 |
|  | Services | 0.02 | 0.00 | 0.00 | 0.00 | 1.00 | 1.00 | 1.01 |
|  | Other | 0.00 | 0.00 | 0.00 | 0.00 | 1.00 | 1.02 | 1.06 |
|  | Remaining components | 0.02 | -0.01 | 0.00 | 0.00 | 1.00 | 1.06 | 1.05 |

# Biodiversity footprints per serving for meat-based and vegetarian meal options in Germany and Indonesia per clustered contributing processes

**Table S10:** Biodiversity footprints per serving for meat-based and vegetarian meal options in Germany and Indonesia (2015 baseline, absolute scenario differences for 2050 and the scenarios SSP1, SSP2, SSP3 and inter-scenario variability benchmarked against the lowest 2050 scenario results) per clustered contributing processes

|  | **Contributing processes** | **Baseline** | **SSP1** | **SSP2** | **SSP3** | **SSP1** | **SSP2** | **SSP3** |
| --- | --- | --- | --- | --- | --- | --- | --- | --- |
|  |  | **2015** | **2050** | | | **2050** | | |
|  |  | [BII loss m^2^ per serving] | Absolute difference to baseline | | | inter-scenario variability (1=min 2050 scenario result) | | |
| **DE Meat-based meal 'Spaghetti Bolognese with beef'** | **Total** | **0.69** | **-0.20** | **-0.13** | **-0.11** | **1.00** | **1.15** | **1.18** |
|  | Plant production | 0.67 | -0.20 | -0.13 | -0.11 | 1.00 | 1.14 | 1.18 |
|  | Animal production | 0.00 | 0.00 | 0.00 | 0.00 | 0.00 | 0.00 | 0.00 |
|  | Fertilizers | 0.00 | 0.00 | 0.00 | 0.00 | 1.00 | 1.07 | 1.09 |
|  | Food products | 0.00 | 0.00 | 0.00 | 0.00 | 1.00 | 1.29 | 1.37 |
|  | Energy | 0.00 | 0.00 | 0.00 | 0.00 | 0.00 | 0.00 | 0.00 |
|  | Manufacturing | 0.00 | 0.00 | 0.00 | 0.00 | 0.00 | 0.00 | 0.00 |
|  | Industrial processes | 0.00 | 0.00 | 0.00 | 0.00 | 1.00 | 1.12 | 1.15 |
|  | Services | 0.00 | 0.00 | 0.00 | 0.00 | 0.90 | 0.98 | 1.00 |
|  | Other | 0.00 | 0.00 | 0.00 | 0.00 | 0.00 | 0.00 | 0.00 |
|  | Remaining components | 0.02 | -0.01 | 0.00 | 0.00 | 1.00 | 1.24 | 1.32 |
| **DE Plant-based meal 'Spaghetti Bolognese with lentils'** | **Total** | **0.19** | **-0.03** | **-0.03** | **-0.03** | **1.00** | **1.00** | **1.02** |
|  | Plant production | 0.19 | -0.03 | -0.03 | -0.03 | 1.00 | 1.00 | 1.01 |
|  | Animal production | 0.00 | 0.00 | 0.00 | 0.00 | 0.00 | 0.00 | 0.00 |
|  | Fertilizers | 0.00 | 0.00 | 0.00 | 0.00 | 0.00 | 0.00 | 0.00 |
|  | Food products | 0.00 | 0.00 | 0.00 | 0.00 | 0.00 | 0.00 | 0.00 |
|  | Energy | 0.00 | 0.00 | 0.00 | 0.00 | 0.00 | 0.00 | 0.00 |
|  | Manufacturing | 0.00 | 0.00 | 0.00 | 0.00 | 0.00 | 0.00 | 0.00 |
|  | Industrial processes | 0.00 | 0.00 | 0.00 | 0.00 | 1.00 | 1.00 | 1.03 |
|  | Services | 0.00 | 0.00 | 0.00 | 0.00 | 0.00 | 0.00 | 0.00 |
|  | Other | 0.00 | 0.00 | 0.00 | 0.00 | 0.00 | 0.00 | 0.00 |
|  | Remaining components | 0.00 | 0.00 | 0.00 | 0.00 | 1.00 | 1.01 | 1.22 |

|  | **Contributing processes** | **Baseline** | **SSP1** | **SSP2** | **SSP3** | **SSP1** | **SSP2** | **SSP3** |
| --- | --- | --- | --- | --- | --- | --- | --- | --- |
|  |  | **2015** | **2050** | | | **2050** | | |
|  |  | [BII loss m^2^ per serving] | Absolute difference to baseline | | | inter-scenario variability (1=min 2050 scenario result) | | |
| **I ID Meat-based meal 'Chicken soup, fried beans, rice, fruit'** | **Total** | **0.38** | **-0.10** | **-0.09** | **-0.08** | **1.00** | **1.04** | **1.08** |
|  | Plant production | 0.37 | -0.10 | -0.09 | -0.08 | 1.00 | 1.04 | 1.08 |
|  | Animal production | 0.00 | 0.00 | 0.00 | 0.00 | 0.00 | 0.00 | 0.00 |
|  | Fertilizers | 0.00 | 0.00 | 0.00 | 0.00 | 0.00 | 0.00 | 0.00 |
|  | Food products | 0.01 | 0.00 | 0.00 | 0.00 | 1.00 | 1.07 | 1.11 |
|  | Energy | 0.00 | 0.00 | 0.00 | 0.00 | 0.00 | 0.00 | 0.00 |
|  | Manufacturing | 0.00 | 0.00 | 0.00 | 0.00 | 0.00 | 0.00 | 0.00 |
|  | Industrial processes | 0.00 | 0.00 | 0.00 | 0.00 | 1.00 | 1.01 | 1.04 |
|  | Services | 0.00 | 0.00 | 0.00 | 0.00 | 0.00 | 0.00 | 0.00 |
|  | Other | 0.00 | 0.00 | 0.00 | 0.00 | 0.00 | 0.00 | 0.00 |
|  | Remaining components | 0.01 | 0.00 | 0.00 | 0.00 | 1.00 | 1.01 | 1.07 |
| **I ID Plant-based meal 'Tofu soup, omelet, rice, fruit'** | **Total** | **0.26** | **-0.07** | **-0.07** | **-0.06** | **1.00** | **1.01** | **1.05** |
|  | Plant production | 0.25 | -0.07 | -0.06 | -0.06 | 1.00 | 1.01 | 1.05 |
|  | Animal production | 0.00 | 0.00 | 0.00 | 0.00 | 1.00 | 1.04 | 1.06 |
|  | Fertilizers | 0.00 | 0.00 | 0.00 | 0.00 | 0.00 | 0.00 | 0.00 |
|  | Food products | 0.00 | 0.00 | 0.00 | 0.00 | 1.00 | 1.07 | 1.11 |
|  | Energy | 0.00 | 0.00 | 0.00 | 0.00 | 0.00 | 0.00 | 0.00 |
|  | Manufacturing | 0.00 | 0.00 | 0.00 | 0.00 | 0.00 | 0.00 | 0.00 |
|  | Industrial processes | 0.00 | 0.00 | 0.00 | 0.00 | 1.00 | 1.00 | 1.02 |
|  | Services | 0.00 | 0.00 | 0.00 | 0.00 | 0.00 | 0.00 | 0.00 |
|  | Other | 0.00 | 0.00 | 0.00 | 0.00 | 0.00 | 0.00 | 0.00 |
|  | Remaining components | 0.00 | 0.00 | 0.00 | 0.00 | 1.00 | 1.01 | 1.04 |

|  | **Contributing processes** | **Baseline** | **SSP1** | **SSP2** | **SSP3** | **SSP1** | **SSP2** | **SSP3** |
| --- | --- | --- | --- | --- | --- | --- | --- | --- |
|  |  | **2015** | **2050** | | | **2050** | | |
|  |  | [BII loss m^2^ per serving] | Absolute difference to baseline | | | inter-scenario variability (1=min 2050 scenario result) | | |
| **ID Meat-based meal 'Fried beans'** | **Total** | **0.05** | **0.00** | **0.00** | **0.00** | **1.00** | **1.01** | **1.01** |
|  | Plant production | 0.04 | 0.00 | 0.00 | 0.00 | 1.00 | 1.01 | 1.01 |
|  | Animal production | 0.00 | 0.00 | 0.00 | 0.00 | 0.00 | 0.00 | 0.00 |
|  | Fertilizers | 0.00 | 0.00 | 0.00 | 0.00 | 0.00 | 0.00 | 0.00 |
|  | Food products | 0.00 | 0.00 | 0.00 | 0.00 | 1.00 | 1.07 | 1.11 |
|  | Energy | 0.00 | 0.00 | 0.00 | 0.00 | 0.00 | 0.00 | 0.00 |
|  | Manufacturing | 0.00 | 0.00 | 0.00 | 0.00 | 0.00 | 0.00 | 0.00 |
|  | Industrial processes | 0.00 | 0.00 | 0.00 | 0.00 | 1.00 | 1.00 | 1.01 |
|  | Services | 0.00 | 0.00 | 0.00 | 0.00 | 0.00 | 0.00 | 0.00 |
|  | Other | 0.00 | 0.00 | 0.00 | 0.00 | 0.00 | 0.00 | 0.00 |
|  | Remaining components | 0.00 | 0.00 | 0.00 | 0.00 | 1.00 | 1.02 | 1.06 |
| **ID Plant-based meal 'Omelet'** | **Total** | **0.15** | **-0.04** | **-0.04** | **-0.03** | **1.00** | **1.05** | **1.12** |
|  | Plant production | 0.14 | -0.04 | -0.04 | -0.03 | 1.00 | 1.05 | 1.12 |
|  | Animal production | 0.00 | 0.00 | 0.00 | 0.00 | 1.00 | 1.04 | 1.06 |
|  | Fertilizers | 0.00 | 0.00 | 0.00 | 0.00 | 0.00 | 0.00 | 0.00 |
|  | Food products | 0.00 | 0.00 | 0.00 | 0.00 | 1.00 | 1.07 | 1.11 |
|  | Energy | 0.00 | 0.00 | 0.00 | 0.00 | 0.00 | 0.00 | 0.00 |
|  | Manufacturing | 0.00 | 0.00 | 0.00 | 0.00 | 0.00 | 0.00 | 0.00 |
|  | Industrial processes | 0.00 | 0.00 | 0.00 | 0.00 | 0.00 | 0.00 | 0.00 |
|  | Services | 0.00 | 0.00 | 0.00 | 0.00 | 0.00 | 0.00 | 0.00 |
|  | Other | 0.00 | 0.00 | 0.00 | 0.00 | 0.00 | 0.00 | 0.00 |
|  | Remaining components | 0.00 | 0.00 | 0.00 | 0.00 | 1.00 | 1.04 | 1.11 |

|  | **Contributing processes** | **Baseline** | **SSP1** | **SSP2** | **SSP3** | **SSP1** | **SSP2** | **SSP3** |
| --- | --- | --- | --- | --- | --- | --- | --- | --- |
|  |  | **2015** | **2050** | | | **2050** | | |
|  |  | [BII loss m^2^ per serving] | Absolute difference to baseline | | | inter-scenario variability (1=min 2050 scenario result) | | |
| **ID Meat-based meal 'Chicken soup'** | **Total** | **0.26** | **-0.08** | **-0.07** | **-0.06** | **1.00** | **1.06** | **1.14** |
|  | Plant production | 0.25 | -0.08 | -0.07 | -0.06 | 1.00 | 1.06 | 1.14 |
|  | Animal production | 0.00 | 0.00 | 0.00 | 0.00 | 0.00 | 0.00 | 0.00 |
|  | Fertilizers | 0.00 | 0.00 | 0.00 | 0.00 | 0.00 | 0.00 | 0.00 |
|  | Food products | 0.00 | 0.00 | 0.00 | 0.00 | 1.00 | 1.07 | 1.11 |
|  | Energy | 0.00 | 0.00 | 0.00 | 0.00 | 0.00 | 0.00 | 0.00 |
|  | Manufacturing | 0.00 | 0.00 | 0.00 | 0.00 | 0.00 | 0.00 | 0.00 |
|  | Industrial processes | 0.00 | 0.00 | 0.00 | 0.00 | 1.00 | 1.05 | 1.11 |
|  | Services | 0.00 | 0.00 | 0.00 | 0.00 | 0.00 | 0.00 | 0.00 |
|  | Other | 0.00 | 0.00 | 0.00 | 0.00 | 0.00 | 0.00 | 0.00 |
|  | Remaining components | 0.00 | 0.00 | 0.00 | 0.00 | 1.00 | 1.11 | 1.22 |
| **ID Plant-based meal 'Tofu soup'** | **Total** | **0.03** | **-0.01** | **-0.01** | **-0.01** | **1.05** | **1.00** | **1.01** |
|  | Plant production | 0.03 | -0.01 | -0.01 | -0.01 | 1.05 | 1.00 | 1.01 |
|  | Animal production | 0.00 | 0.00 | 0.00 | 0.00 | 0.00 | 0.00 | 0.00 |
|  | Fertilizers | 0.00 | 0.00 | 0.00 | 0.00 | 0.00 | 0.00 | 0.00 |
|  | Food products | 0.00 | 0.00 | 0.00 | 0.00 | 0.00 | 0.00 | 0.00 |
|  | Energy | 0.00 | 0.00 | 0.00 | 0.00 | 0.00 | 0.00 | 0.00 |
|  | Manufacturing | 0.00 | 0.00 | 0.00 | 0.00 | 0.00 | 0.00 | 0.00 |
|  | Industrial processes | 0.00 | 0.00 | 0.00 | 0.00 | 1.00 | 1.00 | 1.02 |
|  | Services | 0.00 | 0.00 | 0.00 | 0.00 | 0.00 | 0.00 | 0.00 |
|  | Other | 0.00 | 0.00 | 0.00 | 0.00 | 0.00 | 0.00 | 0.00 |
|  | Remaining components | 0.00 | 0.00 | 0.00 | 0.00 | 1.00 | 1.03 | 1.08 |

# Indonesian Meal component climate and biodiversity footprints

## Soup

**
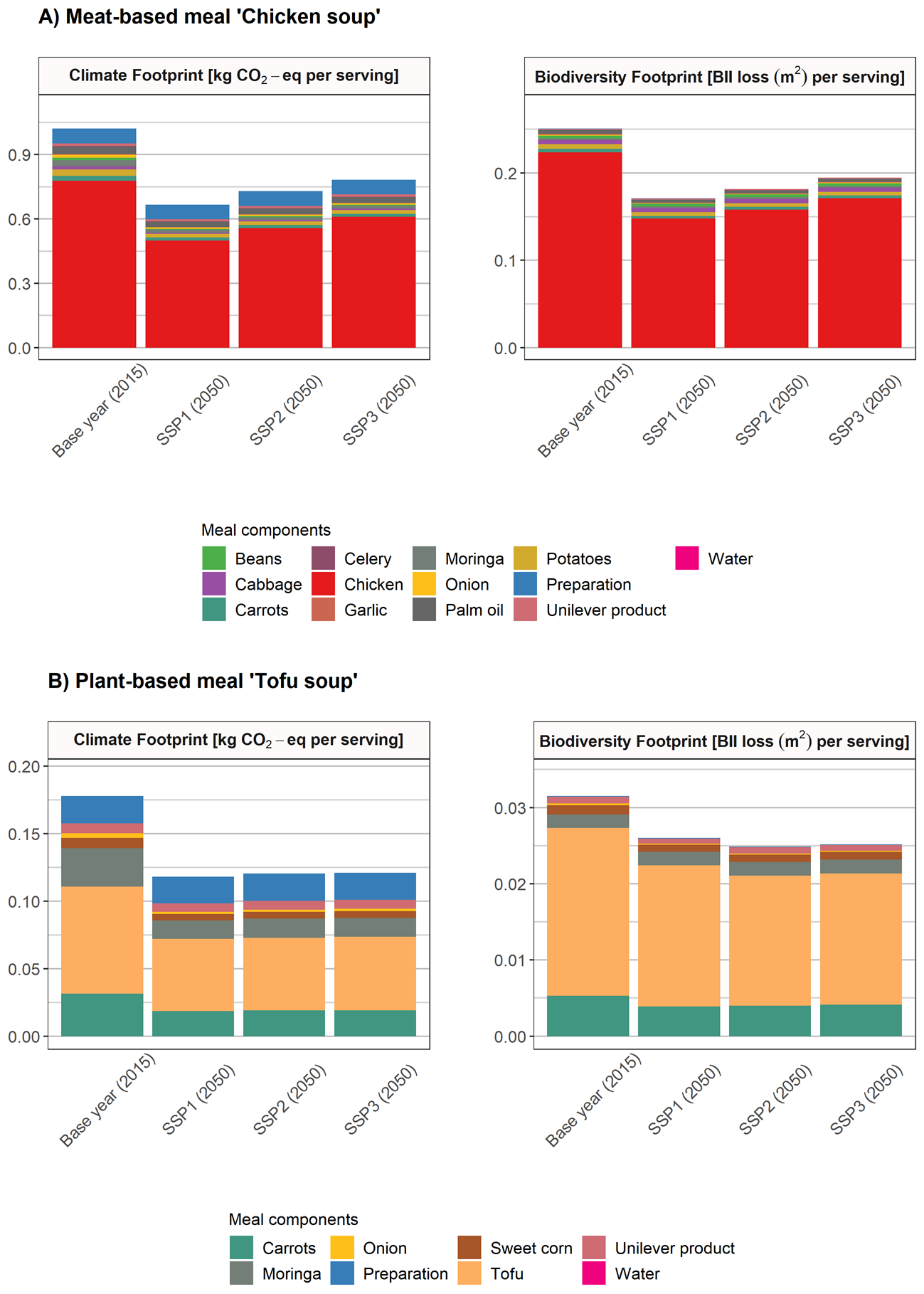
**

**Fig. S2.** Climate and biodiversity footprints per serving for meat-based and plant-based meal options in Indonesia for the 2015 Base year and the 2050 SSP1, SSP2 and SSP3 scenarios: A) Meat-based meal ‘Chicken soup’ B) Plant-based meal ‘Tofu soup’.

## Side dish


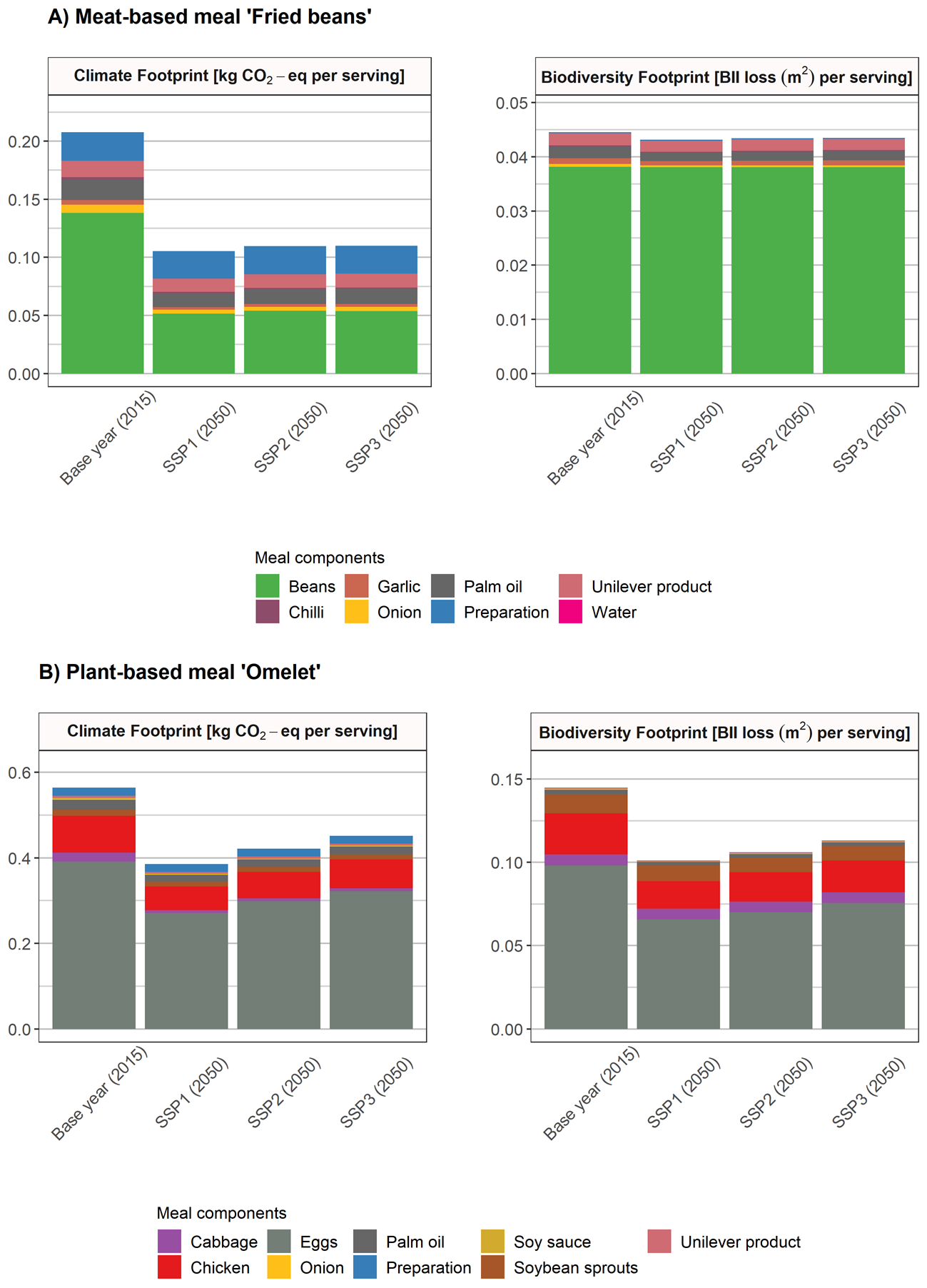


**Fig. S3.** Climate and biodiversity footprints per serving for meat-based and plant-based meal options in Indonesia for the 2015 Base year and the 2050 SSP1, SSP2 and SSP3 scenarios: A) Meat-based meal ‘Fried beans’ B) Plant-based meal ‘Omelet’.

# German climate and biodiversity footprints compared to baseline results – Response to types of changes


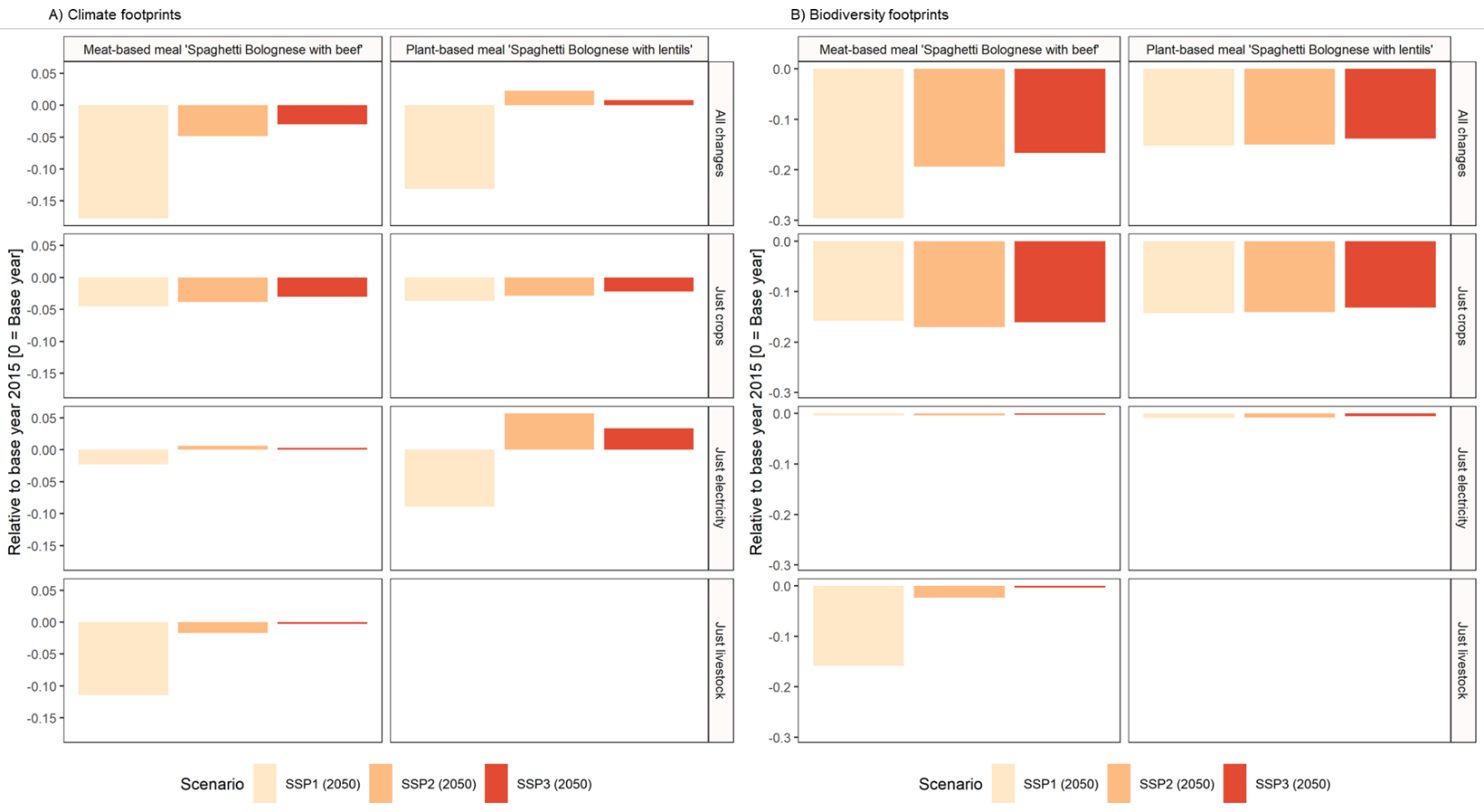


**Fig. S4.** Germany – Meal climate and biodiversity footprint results relative to baseline assuming implementation of all prospective changes (crops, electricity, livestock), just crops, just electricity, just livestock – A) Climate footprints B) Biodiversity footprints.

# Indonesian climate and biodiversity footprints (main meal) compared to baseline results – Response to types of changes


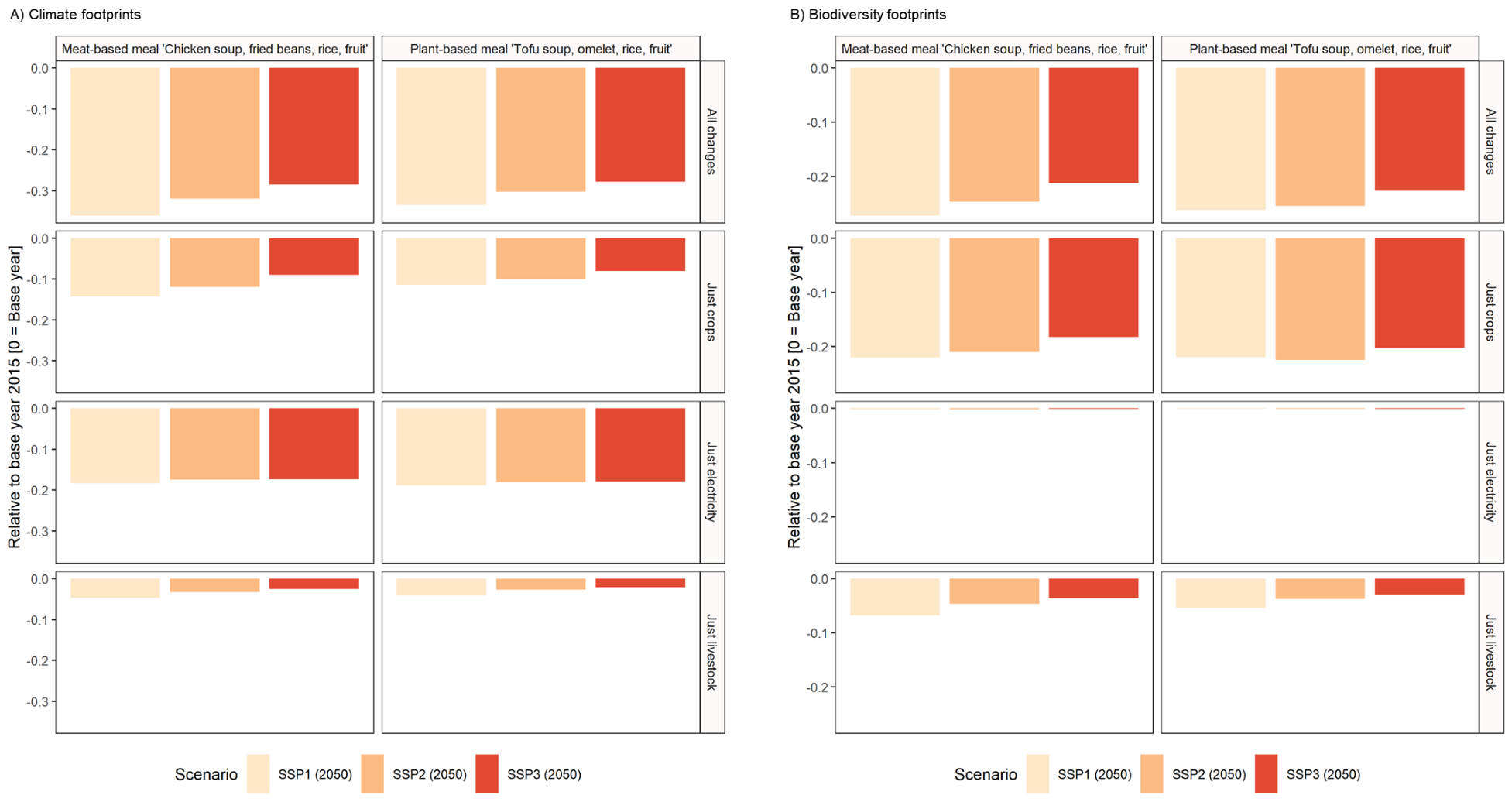


**Fig. S5.** Indonesia - Meal climate and biodiversity footprint results relative to baseline assuming implementation of all prospective changes (crops, electricity, livestock), just crops, just electricity, just livestock – A) Climate footprints B) Biodiversity footprints.

# Climate and biodiversity footprints per serving and per 100 kcal

**Table S11:** Climate and biodiversity footprints per serving and per 100kcal for meat-based and vegetarian meal options in A) Germany and B) Indonesia (2015 baseline, 2050 values for the scenarios SSP1, SSP2 and SSP3)

**A) Germany**

|  |  |  |  |  |  |  |
| --- | --- | --- | --- | --- | --- | --- |
|  |  |  | **Climate footprint**  **[kg CO_2_-eq]** | | **Biodiversity footprint**  **[BII loss m^2^]** | |
|  |  |  | per serving | per 100kcal | per serving | per 100kcal |
| **Meat-based meal 'Spaghetti Bolognese with beef'** | **Baseline** | **2015** | **4.04** | **0.798** | **0.69** | **0.136** |
|  | SSP1 | 2050 | 3.32 | 0.656 | 0.48 | 0.096 |
|  | SSP2 |  | 3.84 | 0.759 | 0.56 | 0.110 |
|  | SSP3 |  | 3.91 | 0.774 | 0.57 | 0.113 |
| **Plant-based meal 'Spaghetti Bolognese with lentils'** | **Baseline** | **2015** | **0.70** | **0.133** | **0.19** | **0.036** |
|  | SSP1 | 2050 | 0.61 | 0.116 | 0.16 | 0.031 |
|  | SSP2 |  | 0.72 | 0.136 | 0.16 | 0.031 |
|  | SSP3 |  | 0.71 | 0.134 | 0.17 | 0.031 |

**B) Indonesia**

|  |  |  | **Climate footprint**  **[kg CO_2_-eq]** | | **Biodiversity footprint**  **[BII loss m^2^]** | |
| --- | --- | --- | --- | --- | --- | --- |
|  |  |  | per serving | per 100kcal | per serving | per 100kcal |
| **Meat-based meal 'Chicken soup, fried beans, rice, fruit'** | **Baseline** | **2015** | **1.60** | **0.181** | **0.37** | **0.042** |
|  | SSP1 | 2050 | 1.02 | 0.116 | 0.27 | 0.031 |
|  | SSP2 |  | 1.09 | 0.123 | 0.28 | 0.032 |
|  | SSP3 |  | 1.15 | 0.130 | 0.30 | 0.033 |
| **Plant-based meal 'Tofu soup, omelet, rice, fruit'** | **Baseline** | **2015** | **1.28** | **0.253** | **0.26** | **0.051** |
|  | SSP1 | 2050 | 0.85 | 0.168 | 0.19 | 0.038 |
|  | SSP2 |  | 0.89 | 0.176 | 0.19 | 0.038 |
|  | SSP3 |  | 0.92 | 0.183 | 0.20 | 0.039 |
